# Targeting the permeability barrier and peptidoglycan recycling pathways to disarm *Pseudomonas aeruginosa* against the innate immune system

**DOI:** 10.1371/journal.pone.0181932

**Published:** 2017-07-25

**Authors:** Gabriel Torrens, Marcelo Pérez-Gallego, Bartolomé Moya, Marta Munar-Bestard, Laura Zamorano, Gabriel Cabot, Jesús Blázquez, Juan A. Ayala, Antonio Oliver, Carlos Juan

**Affiliations:** 1 Servicio de Microbiología and Unidad de Investigación, Hospital Son Espases, Instituto de Investigación Sanitaria de Baleares (IdISBa), Palma, Spain; 2 Departamento de Biotecnología Microbiana, Centro Nacional de Biotecnología, Madrid, Spain; 3 Departamento de Virología y Microbiología, Centro de Biología Molecular Severo Ochoa, Madrid, Spain; Pusan National University, REPUBLIC OF KOREA

## Abstract

Antimicrobial resistance is a continuously increasing threat that severely compromises our antibiotic arsenal and causes thousands of deaths due to hospital-acquired infections by pathogens such as *Pseudomonas aeruginosa*, situation further aggravated by the limited development of new antibiotics. Thus, alternative strategies such as those targeting bacterial resistance mechanisms, virulence or potentiating the activity of our immune system resources are urgently needed. We have recently shown that mutations simultaneously causing the peptidoglycan recycling blockage and the β-lactamase AmpC overexpression impair the virulence of *P*.*aeruginosa*. These findings suggested that peptidoglycan metabolism might be a good target not only for fighting antibiotic resistance, but also for the attenuation of virulence and/or potentiation of our innate immune weapons. Here we analyzed the activity of the innate immune elements peptidoglycan recognition proteins (PGRPs) and lysozyme against *P*. *aeruginosa*. We show that while lysozyme and PGRPs have a very modest basal effect over *P*. *aeruginosa*, their bactericidal activity is dramatically increased in the presence of subinhibitory concentrations of the permeabilizing agent colistin. We also show that the *P*. *aeruginosa* lysozyme inhibitors seem to play a very residual protective role even in permeabilizing conditions. In contrast, we demonstrate that, once the permeability barrier is overpassed, the activity of lysozyme and PGRPs is dramatically enhanced when inhibiting key peptidoglycan recycling components (such as the 3 AmpDs, AmpG or NagZ), indicating a decisive protective role for cell-wall recycling and that direct peptidoglycan-binding supports, at least partially, the activity of these enzymes. Finally, we show that recycling blockade when occurring simultaneously with AmpC overexpression determines a further decrease in the resistance against PGRP2 and lysozyme, linked to quantitative changes in the cell-wall. Thus, our results help to delineate new strategies against *P*. *aeruginosa* infections, simultaneously targeting β–lactam resistance, cell-wall metabolism and virulence, ultimately enhancing the activity of our innate immune weapons.

## Introduction

*Pseudomonas aeruginosa* is a paradigmatic example of adaptable microorganism thanks to its outsized metabolic plasticity and versatility [1,2]. It is a major opportunistic pathogen, being one of the first causes of nosocomial infections, particularly in critically ill and immunocompromised patients [3]. *P*. *aeruginosa* is the top pathogen causing ventilator-associated pneumonia and burn wound infections, and a major cause of nosocomial bacteremia [3,4]. It is the most frequent driver of chronic respiratory infections in patients with cystic fibrosis or other chronic underlying diseases [5].

One of the most striking characteristics of *P*. *aeruginosa* is its outstanding capacity for antibiotic resistance development through chromosomal mutations and/or acquisition of horizontally transmitted determinants [6]. Among *P*. *aeruginosa* β-lactam resistance mechanisms, particularly noteworthy is the chromosomal β-lactamase AmpC, whose regulation is intimately linked to the peptidoglycan recycling [7]. Mutation of different peptidoglycan recycling components (such as AmpD amidases) leads to a stepwise upregulation of the β-lactamase, frequently causing clinical resistance to the antipseudomonal β-lactams [8]. Moreover, the inhibition of other peptidoglycan recycling components, such as AmpG or NagZ, has been demonstrated to mitigate β-lactam and fosfomycin resistance in *P*. *aeruginosa* [9–11]. Thus, peptidoglycan recycling is envisaged as a candidate target for combating *P*. *aeruginosa* resistance [12,13].

Beyond the antibiotic resistance, bacterial virulence/pathogenesis has been proposed as an attractive target for improving the outcome of severe infections and/or facilitating the activity of our innate immune system [14]. Moreover, many evidences of an inverse correlation between resistance and virulence have been described [15,16]. Peptidoglycan recycling is an illustrative model, since we have recently shown that mutations simultaneously leading to the blockage of peptidoglycan recycling and AmpC derepression impair *P*. *aeruginosa* fitness and virulence [17]. In this sense, a very limited number of works describe mutations affecting the peptidoglycan metabolism and cell viability, and almost always referring to gram-positives [18–20]. It has been largely known that the gram-positive cell-wall has a major defensive role, on the contrary of gram-negatives’ peptidoglycan, thinner and protected thanks to the outer membrane [21]. Thus, the search for targets that could impair the resistance of peptidoglycan from gram-negatives is an almost unexplored field.

Among the effectors believed to target the cell-wall, we could highlight the innate immune elements peptidoglycan recognition proteins (PGRPs) and lysozyme. Both had been classically thought to bind and degrade peptidoglycan to exert at least a notable part of their bactericidal effect, making the cell more susceptible to osmotic pressure [22–25]. Mammals have four PGRPs, PGRP1, 2, 3, and 4, being the PGRP1, PGRP3 and PGRP4 thought to be bactericidal through a complex suicide mechanism [22,26–28]. Meanwhile, PGRP2 is an N-acetylmuramoyl-L-alanine amidase that hydrolyzes peptidoglycan between the sugar backbone and the peptide chain, initially described as a scavenger protein intended to reduce the inflammatory capacity of peptidoglycan fragments [29,30]. Regarding the lysozyme, three major types have been identified: the c-type (chicken or conventional-type), the g-type (goose-type) and the i-type (invertebrate type) [31]. It has been suggested that the digestion of peptidoglycan by lysozyme is important to reduce its pro-inflammatory power [32], similarly to PGRP2 activity [33]. Some works have suggested that the c-type lysozyme alleged bactericidal power does not entirely rely on its muramidase activity [34], but also on the capacity to cause perturbation of membranes. The lysozyme hydrolyzes the glycosidic bond between β(1–4)-linked N-acetylmuramic acid and N-acetylglucosamine, but the outer membrane and lipopolysaccharide of gram-negatives seem to play a highly protective role against it. However, some gram-negatives have lysozyme inhibitors to prevent the peptidoglycan degradation in case of membrane permeabilization by mutation and/or by chemical/physical stresses (such as some immune compounds). The two main inhibitors are the Ivy (inhibitor of vertebrate lysozyme) and MliC (membrane bound lysozyme inhibitor of c-type lysozyme) proteins [35]. Both have been shown to notably contribute to *E*. *coli* lysozyme resistance [36–38] as well as the MliC variants of some species of *Salmonella* and *Yersinia* [35,39]. It has been shown that the Ivy (IvyP1) protein from *P*. *aeruginosa* shows a weaker in vitro inhibitory capacity than that of *E*. *coli*, and that the mutant lacking this inhibitor is not more susceptible to lysozyme [37,38]. Interestingly, only certain pseudomonads such as *P*. *aeruginosa*, show a second paralog of IvyP1, called IvyP2. Although the purified IvyP2 has not inhibitory capacity in vitro, its role in vivo has not been ruled out [40]. The potential MliC protective role in *P*. *aeruginosa* has not been demonstrated either [35].

Thus, given the current gaps in our knowledge, this work aimed to understand the activity of the innate immune elements PGRPs and lysozyme against *P*. *aeruginosa* and explore the role of the permeability barrier and peptidoglycan recycling in the process. We show that lysozyme and PGRPs have a very modest effect in regular conditions, but their antipseudomonal power is dramatically increased in the presence of subinhibitory concentrations of a permeabilizing agent such as colistin, which would suggest a direct interaction with peptidoglycan to exert their activity. Our results also suggest that the lysozyme inhibitors of *P*. *aeruginosa* do not play any essential protective role even in permeabilizing conditions. Additionally, our results clearly demonstrate that the peptidoglycan recycling exerts a major protective role against the action of PGRPs and lysozyme when the permeability barrier is overpassed, and that cell-wall recycling blockade together with AmpC de-repression determines a further decrease in the resistance against PGRP2 and lysozyme, linked to quantitative changes in the cell-wall. Taken together our results determine a major step forward for understanding *P*. *aeruginosa* strategies to resist the action of the immune proteins targeting the peptidoglycan, revealing weak points helpful for guiding the development of strategies to fight *P*. *aeruginosa* infections, simultaneously targeting β–lactam resistance, cell-wall metabolism and virulence, ultimately enhancing the activity of some of our immune weapons such as lysozyme and PGRPs.

## Materials and methods

### Bacterial strains and plasmids

The list and description of the strains and plasmids used in this work is shown in the online data supplement (S2 Table). *P*. *aeruginosa* single or combined knockout mutants in *ampD*, *ampDh2*, *ampDh3*, *ampC*, *nagZ* and *ampG* were constructed according to previously described procedures [8] based on the Cre-lox system for gene deletion. The previously constructed plasmid pUCPAD was used for the complementation of selected mutants through electroporation followed by selection in 50 mg/L gentamicin Müller-Hinton agar plates [8]. Plasmid pUCPAC was also used to achieve high levels of AmpC production in selected strains. Additionally, selected mutants from the transposon insertion mutants library by Liberati et al [41] were also used: those in genes MliC (PA14_53040), IvyP1 (PA14_13420), and IvyP2 (PA14_72360) [40]. Minimal inhibitory concentrations (MICs) of colistin for selected strains were determined by E-test (BioMeriéux) in Müller-Hinton agar plates.

### Lysozyme susceptibility assays

The bactericidal activity of chicken egg white lysozyme (circa. 50000 units/mg protein; powder >99% protein) (Sigma-Aldrich) was assessed on selected strains following previously described protocols, with slight modifications [37,38,42]. Approximately 1x10^6^ stationary phase CFUs of each strain were incubated in sodium phosphate buffer (10 mM [pH 7.0]) with 25 mg/L of lysozyme (in a volume of reaction of 0.3 mL) for 1 h at 37°C-180 rpm agitation, and quantified by serial plating on LB agar plates in the beginning and in the end of incubation. The experiments were also performed with the addition of colistin (Sigma-Aldrich) as permeabilizing agent, at a final sub-inhibitory concentration of 0.1 mg/L, in independent experiments. The effect of colistin alone on the different strains was also studied, using the same procedure and buffer without adding lysozyme. All the experiments were performed in quintuplicate.

### PGRPs susceptibility assays

The bactericidal activity of purified human PGRP1, 2, 3 or 4, purchased from AmsBio, was assessed on selected strains following previously described procedures, with slight modifications [27]. Approximately 1x10^5^ stationary phase CFUs were incubated 2h at 180rpm-37°C in the assay buffer: 50 mg/L of the corresponding PGRP, 5 mM Tris-HCl buffer (pH 7.6), containing NaCl 150 mM, ZnSO4 5 μM, Glycerol 5% and LB broth 1%. The viable bacteria were quantified by serial plating on LB agar plates in the beginning and in the end of incubation. The experiments were also performed with the addition of colistin, at 0.1 mg/L, in independent experiments. The effect of colistin alone on the different strains was also studied, using the same procedure and buffers without the addition of any PGRP. All the experiments were performed in quintuplicate.

### Cell-free bacterial supernatants preparation and inactivation of bacteria

Supernatants proceeding from overnight LB cultures of the selected strains were adjusted to an OD600 = 2 with fresh LB, centrifuged to pellet the cells, and finally, filtered through 0.22 μm filters. For bacteria inactivation, appropriate volume aliquots of overnight LB cultures were taken to have an approximate number of 5x10^6^ CFUs. The samples were then centrifuged and resuspended with 20 μL of PBS, and incubated in a water bath for 10 min at 96°C.

### Peptidoglycan purification for NOD receptors stimulation

The peptidoglycans (PGN) from selected mutants were extracted following previously described protocols with slight modifications [43,44]. PAO1, PA14 and derived mutants were cultured overnight in LB broth at 37°C and 180 rpm. The cells were centrifuged and resuspended in double-distilled water. An equal volume of boiling 20% SDS solution was slowly added, and the final suspension was kept boiling for 12 h with stirring. The suspensions were centrifuged at 18000 g for 45 min to collect the sacculi fraction, which was then washed with warm sterile double-distilled water at least three times. PGNs were suspended in 10 ml of 10 mM Tris-HCl (pH 7.6) supplemented with 0.5 mM CaCl_2_ and 2.5 mM MgCl_2_, and treated with 100 μg/ml α-amylase (Sigma-Aldrich), 20 Units of Turbo DNAse (Ambion), 20 Units of RNAse (Sigma-Aldrich) for 2 h at 37°C, and finally with 100 μg/ml of pre-activated pronase E (Merck) at 60°C for 90 min. The enzymes were inactivated by boiling for 20 min in 1% SDS. Next, PGNs were collected and washed as described above. After that, PGNs were lyophilized for weighing and quantification. Samples were then resuspended in 8 M LiCl and incubated for 1 h at room temperature. The PGNs were centrifuged and washed at least three times with double-distilled water, and treated with 100 mM EDTA for 1 h at room temperature. Samples were centrifuged and washed as above, and treated with acetone for 1 h at room temperature. After at least three washes with double-distilled water, the pellets were resuspended in 50 mM NaH_2_PO_4_ (pH 4.9) and digested with 100 μg/ml mutanolysin (Sigma-Aldrich) at 37°C overnight. Next, the enzyme was inactivated by boiling the sample for 10 min and the samples were centrifuged for 5 min to remove insoluble debris. Finally, the supernatants were filtered through 0.22 μm filters to ensure sterility. The E-toxate reagent (Sigma-Aldrich) was used following manufacturer’s instructions to check the absence of endotoxin contamination in the purified PGNs.

### NOD receptors activation

To study the activation levels of NOD1 and NOD2 receptors caused by different stimuli (specified below), the reporter cell lines HEK-Blue hNOD1 and hNOD2 were used, following manufacturer’s instructions (Invivogen). The hNODs activation can be measured in the cited cell lines thanks to the multicopy expression of each NOD variant in the respective line, and the insertion of the secreted embryonic alkaline phosphatase (SEAP) gene under the control of NF-kB and AP-1 (both activated in turn, by NODs). The SEAP secretion is hence proportional to NOD activation, causing the metabolization of the SEAP substrate in the medium, allowing the appearance of blue color which can be spectrophotometrically measured. The hNOD lines were routinely maintained in DMEM (Sigma-Aldrich) supplemented with 10% of heat-inactivated fetal bovine serum, 10mM HEPES, 2 mM L-glutamine and 1X antibiotic-antimycotic solution (Biowest) plus two antibiotics intended for the maintenance of the reporter function-linked plasmids: Blasticidin 30 μg/ml and Zeocin100 μg/ml. Before the experiments, the cells were seeded in 96-well plates (density of approx. 5x10^4^ cells/well), using 180 μL of HEK-Blue detection medium per well. The different stimuli were added afterwards, always in a final volume of 20 μL of PBS per well. After 20 h of stimulation the absorbance at 620 nm was read using a Sinergy H1 microplate reader (Biotek). The stimuli used for these experiments were: i) purified PGNs of PAO1 and derived mutants, 1 μg/well for hNOD1 and 0.25 μg/well for hNOD2; ii) filtered bacterial cultures supernatants prepared as described above (20 μL of supernatant diluted in 180 μL of detection medium); iii) heat-inactivated bacteria in PBS, MOI 1000 for hNOD1 and MOI 500 for hNOD2; and iv) alive bacteria, resuspended in PBS, at a MOI of 250. At least 21 wells per stimulus were used: seven wells from each of 3 independent plates. Positive control wells were always used; hNOD1: 0.2 μg of C12-iE-DAP (Invivogen) in 20 μL of PBS per well, and hNOD2: 1 μg of MDP (Invivogen) in 20 μL of PBS per well. To check the absence of contaminants potentially activating the NF-kB and/or AP-1routes, and to scan the basal level of activation in the cells, negative control wells (20 μL of PBS) were routinely used.

### Preparation of peptidoglycan for HPLC analysis

The peptidoglycan purification protocol was similar to that described above, with some exceptions [43]: RNAse, DNAse, LiCl, EDTA and acetone treatments were omitted. After the resuspension in 50 mM phosphate buffer (pH 4.9), samples were digested with 100 μg/ml Cellosyl muramidase (Hoechst AG, Frankfurt, Germany) at 37°C overnight. The enzyme was then inactivated by 10 min boiling, and centrifuged at 14,000 rpm to remove insoluble debris. The supernatant was mixed with 1/3 volume of 0.5 M sodium borate buffer (pH 9.0) and reduced with excess sodium borohydride (NaBH4) for 30 min at room temperature. The pH was adjusted to pH 3 with orthophosphoric acid. All samples were 0.22-μm filtered and injected into the HPLC. Separations were performed on a Breeze 2 HPLC system, consisting of a 1525 binary HPLC pump model code 5CH (Waters), a UV-visible detector 2489 (Waters), a manual injector model 7725i (Rheodyne), and an Aeris Peptide XB-C18, 3.6 μm, 250 by 4.6 mm reverse-phase column (Phenomenex). Separation of individual components (muropeptides) of peptidoglycan was performed in a linear gradient, the column was equilibrated at 45°C, and the eluted compounds were detected at a wavelength of 204 nm. The mobile-phase (A = 50 mM sodium phosphate [pH 4.35]; B = 75 mM sodium phosphate, 15% methanol [pH 4.95]) gradient consisted of elution at 1.0 ml/min with 100% A for 5 min, followed by a 60-min linear gradient to 0% A/100% B and then 100% B for 5 min.

The identification of individual muropeptides was carried out according to retention time, using a comparison analysis with the retention times of known muropeptides. When a difference was found in the retention time of a particular peak, this peak was purified, and the structure was confirmed or characterized by matrix-assisted laser desorption ionization–time of flight (MALDI-TOF) mass spectrometry with the autoflex spectrometer (Bruker Daltonics). Finally, the relative abundances of muropeptides present in each sample were determined by integrating their respective areas of absorption (Breeze 2, Waters program) and expressed as the molar fraction (mol%) relative to the total content.

### Peptidoglycan quantification

The peptidoglycan from selected strains was quantified by means of the titration of meso-diaminopimelic acid (mDAP) concentration, following previously described protocols [45]. Briefly, the murein sacculi from 250 mL LB broth cultures in late exponential phase (adjusted to an OD600 = 0.8, to normalize the number of cells) were hydrolyzed for 18 hours with HCl 6M at 100°C. Afterwards, the samples were liophilyzed, resuspended in water and treated with ninhydrin reagent (250 mg of ninhydrin disolved in 4 mL of 0.6 M phosphoric acid + 6 ml of acetic acid glacial) for 5 minutes at 100°C. OD436 was measured and concentration of muropeptides was calculated using a mDAP standard curve.

### Data analysis

The GraphPad Prism 5 software was used for graphical representation and statistical analysis. Once checked their Gaussian distribution, the quantitative variables were compared using the unpaired two-tailed Student’s t test. When multiple tests had to be done with the same data set, the One-way ANOVA with post hoc Tukey’s multiple comparison tests were performed. For both types of analysis, a *P* value of <0.001 was considered statistically significant.

## Results & discussion

### Subinhibitory concentrations of colistin enhance *P*. *aeruginosa* susceptibility to lysozyme

We first assessed the role of the permeability barrier on the protection of *P*. *aeruginosa* against lysozyme, as well as the potential lysozyme inhibitory activity of IvyP and MliC proteins of *P*. *aeruginosa*. Table 1 shows PAO1 and PA14 survival rates after treatment during 1h with 25 μg/mL of lysozyme alone or combined with subinhibitory concentrations (0.1 μg/mL) of a well-known membrane-permeabilizing agent, as is the colistin. Minimum inhibitory concentrations (MIC) of colistin, as well as the bacterial survival rates after 1h of treatment with 0.1 μg/mL of colistin in the lysozyme assay buffer are also shown for reference. Moreover, all these parameters were also determined for the transposon knockout mutants in the previously described vertebrate lysozyme inhibitors MliC, IvyP1 and IvyP2.

**Table 1:** Susceptibility to colistin, lysozyme and combined treatment (lysozyme+colistin) of PAO1, PA14, and PA14-derived transposon knockout mutants in the lysozyme inhibitory proteins MliC, IvyP1 or its paralog IvyP2.

Strain	Colistin MIC (μg/mL)	Percentage of bacterial survival after each treatment^a^		
		Colistin (0.1 μg/mL)	Lysozyme (25 μg/mL)	Lysozyme + colistin (25 + 0.1 μg/mL)
PAO1	0.75	45.3 ± 11.2	35.9 ± 8.0	4.1 ± 1.3
PA14	0.25	19.1 ± 3.9	52.3 ± 10.2	3.0 ± 0.62
PA14ΔMliC	0.25	22.5 ± 5.6	45.5 ± 12.3	3.6 ± 1.1
PA14ΔIvyP1	0.25	20.1 ± 4.6	49.5 ± 7.3	2.8 ± 0.93
PA14ΔIvyP2	0.25	19.4 ± 4.1	49.6 ± 14.5	3.4 ± 0.91

^a^Percentage of survival after each treatment respect to the initial inoculum. PAO1 and PA14 survival percentages in the lysozyme assay buffer alone were between 80–90% after incubation (data not shown). Shown values represent the mean of 5 independent experiments per strain ± standard deviation (SD). The differences among the percentages of survival of PA14 and its derived mutants were never statistically significant for each treatment (*P* > 0.05 in the One-way ANOVA test).

As can be observed in Table 1, PA14 showed a slightly higher susceptibility to colistin in comparison with PAO1, whereas this trend was the opposite when considering the susceptibility to lysozyme. In any case, at the used concentrations, the bactericidal activity of lysozyme or colistin was modest for both strains. However, the combination of lysozyme and colistin led to a synergistic effect, decreasing survival by approx. 10-fold compared to each compound alone. On the other hand, MliC, lvyP1 and lvyP2 mutants showed no statistically significant differences in survival after treatment with lysozyme alone, colistin alone or combination of both, compared to the wild-type strain PA14 (Table 1): *P* was always > 0.05 in the One-way ANOVA test.

Our results show that lysozyme at physiological concentrations [23, 46] has a very modest antipseudomonal activity. However, when the permeability barrier is overpassed (through subinhibitory concentrations of colistin), its bactericidal activity is greatly enhanced. To our knowledge this is the first work in which synergy between colistin and lysozyme has been demonstrated. Linked to these results, previous studies using *Enterobacter cloacae* or *Acinetobacter baumannii* have evidenced that colistin resistance modulates lysozyme susceptibility through the modification of the lipopolysaccharide [47]. Whereas in some species the orthologues of IvyP1, IvyP2 and MliC have been shown to be very important for resistance to lysozyme [35–38], our results suggest that in *P*. *aeruginosa* they do not play a relevant protective effect when independently inactivated, even when the permeability barrier is overpassed. Our results might thus be consistent with previous works claiming that the role of Ivy proteins is to control the activity of lytic transglycosylases within the periplasm, and that the inhibitory power over lysozyme is simply a coincidence [40]. Nevertheless, the possibility of a quantitative summation of the inhibitory activities when two or even one of the proteins are still expressed can not be completely discarded. Mainly, considering that no previous works have checked if these inhibitors are constitutively expressed in *P*. *aeruginosa*, or that their expression could be inducible in a fashion that our experimental conditions do not allow to appreciate.

### Extense killing of *P*. *aeruginosa* by PGRPs in the presence of subinhibitory concentrations of colistin

**Fig 1:**
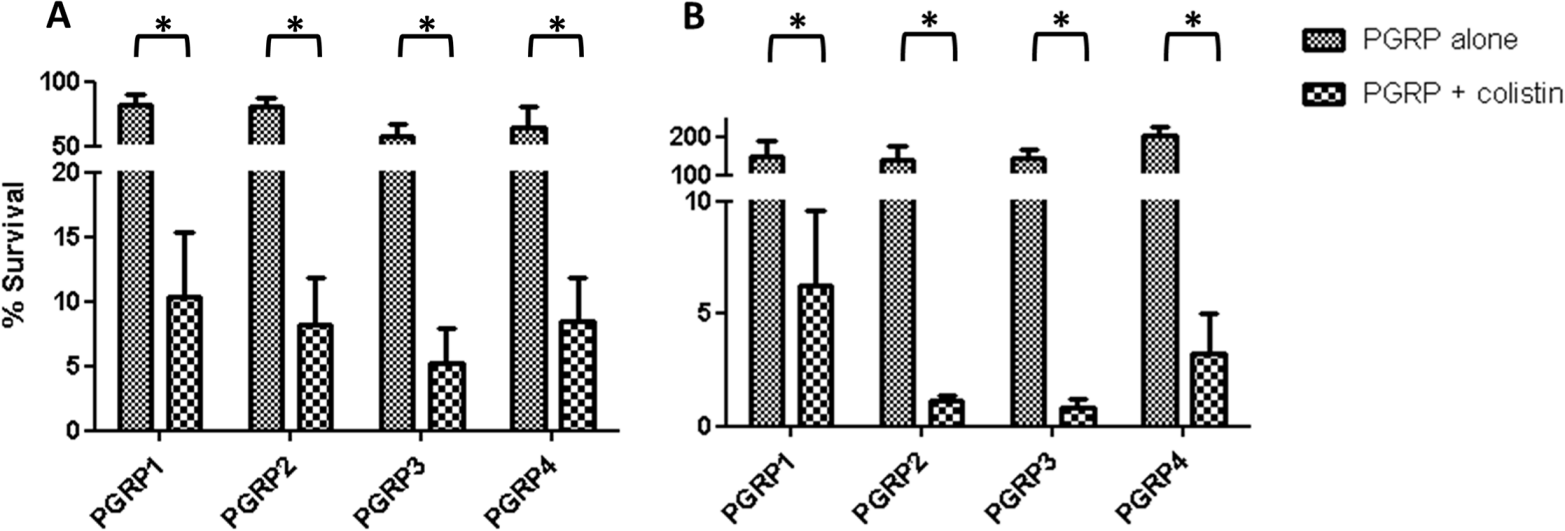
Bactericidal activity of PGRP1, 2, 3 and 4 (50 μg/mL) under regular or membrane permeabilization (colistin 0.1 μg/mL) conditions. The percentages of bacterial survival after treatments respect to the initial inoculum are shown. A) PAO1; B) PA14. The bars represent the mean of 5 experiments and the error bars the SD. *Statistically significant, *P*< 0.001 in the Student’s t-test.

We next assessed the antipseudomonal activity of PGRPs and determined the role of the permeability barrier on the protection of *P*. *aeruginosa* against these innate immune system components. Fig 1 shows the survival rates of PAO1 (A) and PA14 (B) cells treated with PGRP1, PGRP2, PGRP3 and PGRP4, under regular or permeabilization conditions (0.1 μg/mL colistin). The percentages of PAO1 and PA14 survival after incubation with colistin alone in the PGRPs assay buffer were 71.5 ± 10.6 and 59.3 ± 8.6 respectively.

As can be observed, PGRPs produced a very modest reduction in PAO1 bacterial load, with lowest survival (60%) being documented for PGRP3 treated cells. Moreover, none of the PGRPs showed any effect against PA14. The lower anti-pseudomonal activity we have documented compared to previous studies [27,30], especially for PGRP1 and PGRP3, might be related to the slightly lower concentrations used (50 vs 100–150 μg/mL). On the other hand, our results were compatible with previous evidences suggesting that PGRP2 shows no direct bactericidal activity on intact *P*. *aeruginosa* cells [27,30]. However, the addition of subinhibitory concentrations of colistin produced a dramatic enhancement of the activity of all four PGRPs. As shown in Fig 1, survival rates were always below 10% for all combinations of PGRPs with colistin in both strains. The effect for PA14 was even higher than that documented for PAO1, particularly for PGRP2 and PGRP3. Thus, our results demonstrate for the first time that, once overpassed the permeability barrier, PGRP2 exerts a potent antipseudomonal activity, comparable to the rest of PGRPs [48].

Previous studies have suggested that the bactericidal effect of PGRPs is based in lipopolysaccharide and outer membrane binding, activation of two-component systems and suicide after the combination of several types of stress [28]. However, our results suggest that the activity of PGPRs is greatly enhanced once the permeability barrier is overpassed. This fact would suggest a direct interaction of these innate immune proteins with the peptidoglycan to trigger their bactericidal power, a fact that has not been demonstrated yet for gram-negatives, which will be discussed in the next sections.

### Targeting peptidoglycan recycling pathways enables lysozyme and PGRPs to efficiently kill *P*. *aeruginosa*

We next evaluated if, in addition to the permeability barrier, the cell-wall physiology plays any role in the susceptibility to lysozyme and PGRPs. For this purpose we tested a panel of strains defective in different steps of peptidoglycan recycling. The studied strains included: i) The triple amidase (AmpD-AmpDh2-AmpDh3) knockout mutant PAΔDDh2Dh3 showing full AmpC derepression and complete absence of peptidoglycan recycling [17]; the intermediate double amidase mutants, with different levels of AmpC expression and partially impaired recycling, were also included for comparison [17], ii) The NagZ (PAΔnZ) mutant, impairing the N-Acetyl-Muramic Acid and N-Acetyl-Glucosamine recycling pathways, together with the AmpC induction capacity [7] and iii) The AmpG (PAΔAG) mutant, impariring the recycling of all peptidoglycan components, including N-Acetyl-Muramic Acid and N-Acetyl-Glucosamine but also the stem peptides, in addition to blocking AmpC inducibility [7].

**Fig 2:**
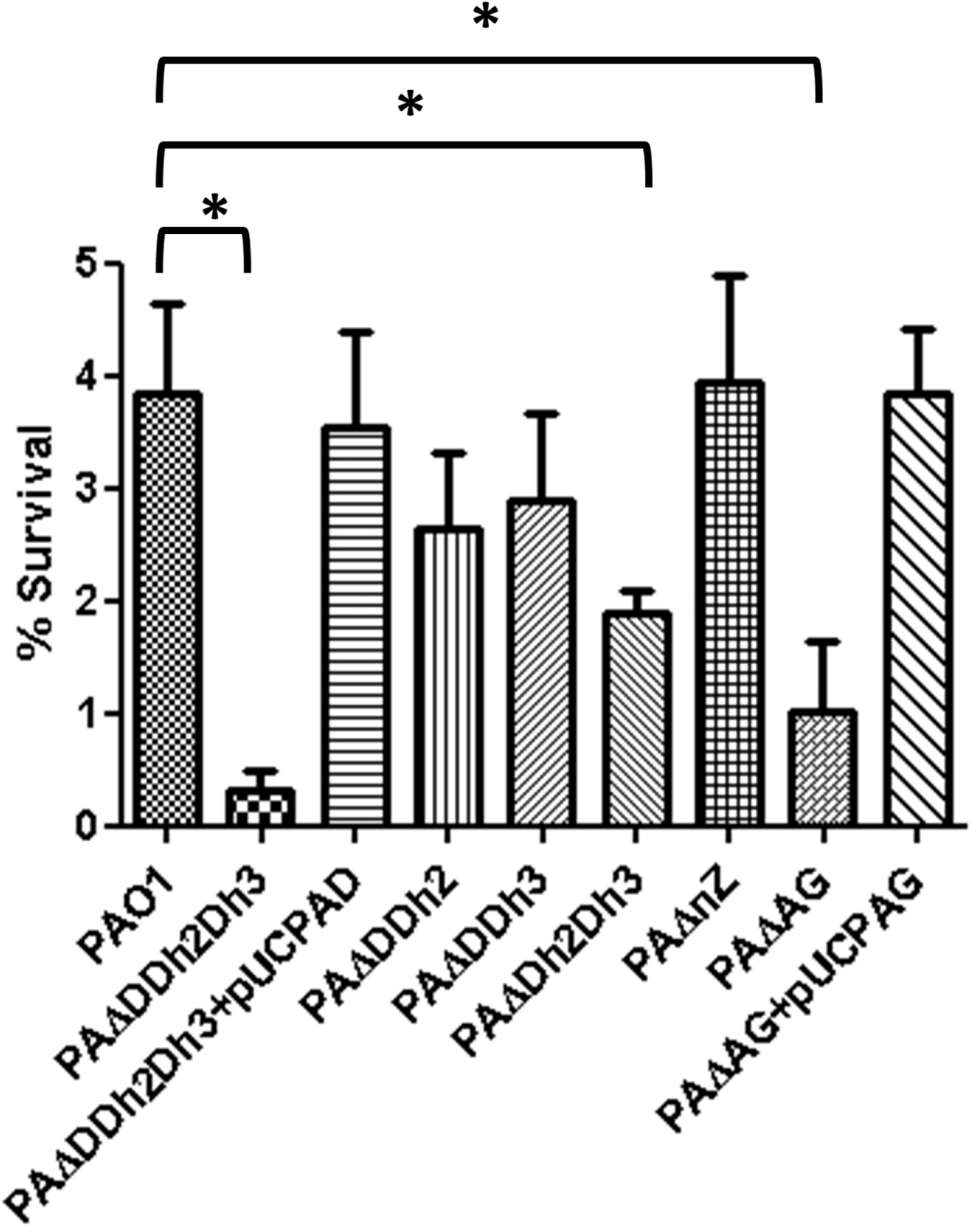
Bactericidal activity of lysozyme (25 μg/mL) + colistin (0.1 μg/mL) combined treatment in PAO1 and derived knockout mutants. The percentages of bacterial survival after treatments respect to the initial inoculum are shown; The bars represent the mean of 5 experiments and the error bars the SD. *Statistically significant, *P*< 0.001 in the One-way ANOVA with post hoc Tukey’s multiple comparison test. PAΔDDh2Dh3: knockout mutant on *ampD*, *ampDh2* and *ampDh3* genes; PAΔnZ: knockout mutant on *nagZ*; PAΔAG: knockout mutant on *ampG*. pUCPAD: pUC18-based *Escherichia-Pseudomonas* shuttle vector containing PAO1 AmpD gene. pUCPAG: pUC18-based *Escherichia-Pseudomonas* shuttle vector containing PAO1 AmpG gene.

The percentages of survival after treatment with lysozyme alone were approximately between 30 and 40% in all the strains, with no statistically significant differences among them (S1 Fig). Likewise, all the strains showed a similar survival rate after incubation with colistin alone in the assay buffer, approximately of 45% (S1 Fig). Thus, the bactericidal effect of lysozyme alone was very modest. However, as shown in Fig 2, synergy with colistin significantly enhanced the bactericidal power of the combined treatment, mainly for several of the mutants. The greatest effect was documented for the triple amidase mutant, showing survival rates of only 0.3%, compared to the 3.9% documented for PAO1. The different double amidase mutants showed a slight decrease in survival respect to PAO1, but only that of AmpDh2-AmpDh3 (1.9%) was statistically significant. No differences in comparison with wild-type were documented when analyzing the survival rates of single mutants (AmpD or AmpDh2 or AmpDh3) (data not shown). As expected, trans-complementation with wild-type *ampD* in PAΔDDh2Dh3 restored wild-type survival rates. Although it did not reach the levels of the tripe amidase mutant, the inactivation of AmpG produced a major increase in lysozyme susceptibility (1.0% survival), which was restored to wildtype levels after transformation with pUCPAG, whereas the inactivation of NagZ did not produce any significant reduction. These results suggested that the recycling of the stem-peptide, not affected in the NagZ mutant, may play a decisive role to mantain the wild type level of protection against lysozyme once permeability barrier is lost. Although lysozyme has been proposed to be bactericidal also independently of its muramidase activity, [34,49], once overpassed the permeability barrier, it should develop its direct lytic activity on the cell-wall. As a peptidoglycan-lytic agent, the ultimate cause of bacterial death after lysozyme treatment would be related with the loss of capacity of the partially degraded peptidoglycan to counteract the osmotic pressure (turgor), leading to cell lysis [21]. Thus, the PAΔDDh2Dh3 hyper-susceptibility to lysozyme + colistin, and to a lesser extent, the increased susceptibility of PAΔAG, could be related with structural defects and/or abnormalities in the cell-wall composition, finally facilitating the action of lysozyme. To test this hypothesis, additional experiments were performed and will be analyzed in the last section.

**Fig 3:**
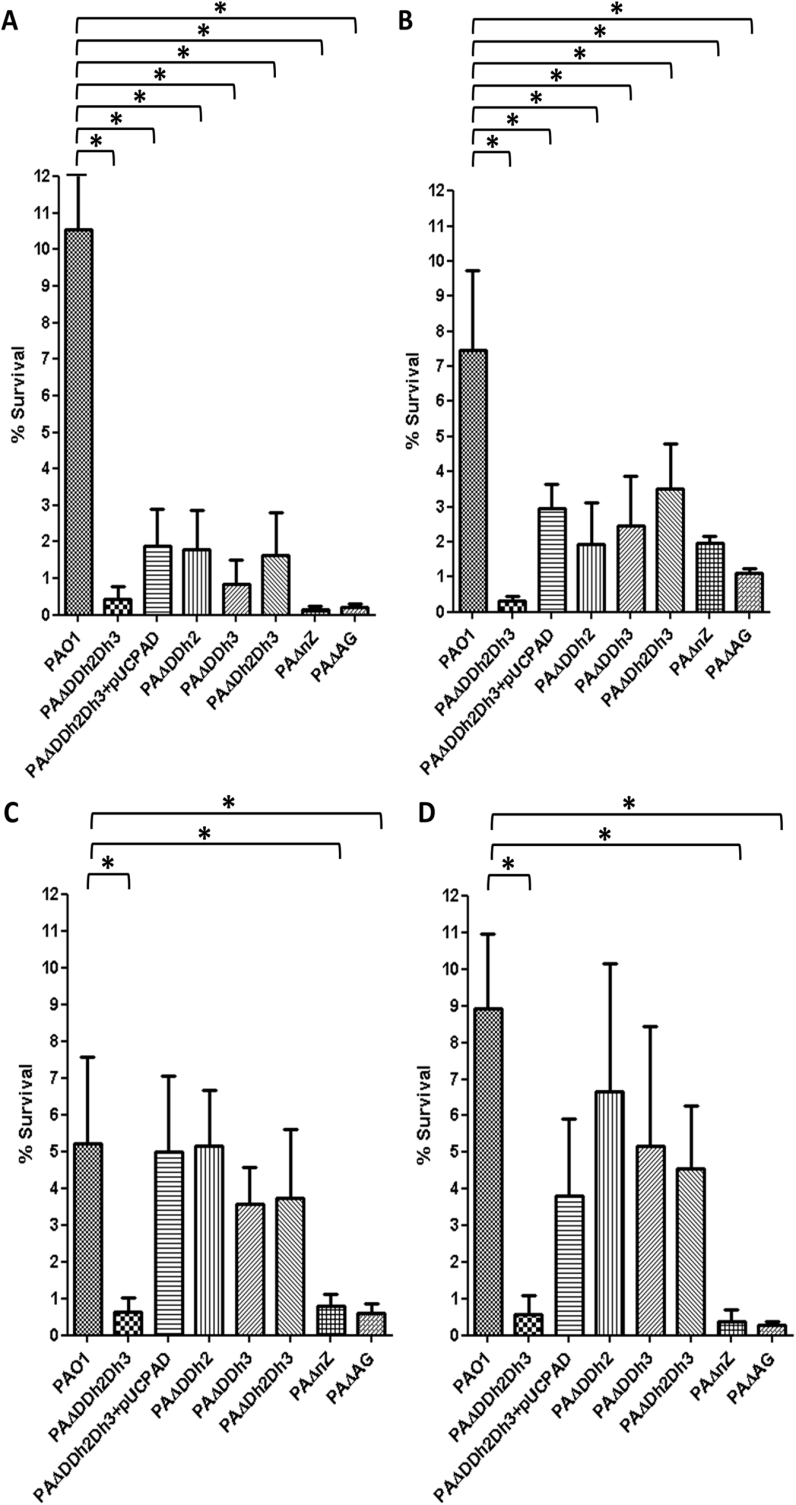
Bactericidal activity of PGRPs (50 μg/mL) + colistin (0.1 μg/mL) combined treatment in PAO1 and derived knockout mutants. **A.** PGRP1. **B.** PGRP2. **C.** PGRP3. **D.** PGRP4. The percentages of bacterial survival after treatments respect to the initial inoculum are shown; The bars represent the mean of 5 experiments and the error bars the SD. *Statistically significant, *P*< 0.001 in the One-way ANOVA with post hoc Tukey’s multiple comparison test. PAΔDDh2Dh3: knockout mutant on *ampD*, *ampDh2* and *ampDh3* genes; PAΔnZ: knockout mutant on *nagZ*; PAΔAG: knockout mutant on *ampG*. pUCPAD: pUC18-based *Escherichia-Pseudomonas* shuttle vector containing PAO1 AmpD gene.

Fig 3 shows the rates of bacterial survival after treatment with PGRPs in the presence of subinhibitory concentrations of colistin for the same panel of mutants in peptidoglycan recycling components. Interestingly, impairment of peptidoglycan recycling at all three levels tested (triple amidase, NagZ and AmpG) produced a marked increase in the bactericidal activity of all four PGRPs. Indeed, for the PGRPs previously considered to be directly bactericidal (1, 3, and 4) [26,28] the increase in activity was equally high for the three types of peptidoglycan recycling mutants, always at least 10-fold higher than that documented for wild-type PAO1, with survival rates well below 1% in all cases. The results for PGRP2 were slightly different, resembling to a certain extent to those obtained for lysozyme. The increase of bactericidal effect was highest for the triple amidase mutant (0.3% survival compared to 7.5% in wild-type), slightly lower in the *ampG* mutant (1.1% of survival) and further lower (although still higher than for wild-type) in the NagZ mutant (2.0% of survival). To confirm the trans-complementation of the phenotypes of increased susceptibility against PGRPs of PAΔAG and PAΔnZ, these mutants were transformed with the wildtype genes cloned in the plasmids pUCPAG and pUCPnZ respectively. As a test for the trans-complementation, these constructs were subjected to the following assays, in which the percentages of survival were i) PGRP1+colistin: 9.5±1.8 for PAΔAG+pUCPAG and 9.1±2.4 for PAΔnZ+pUCPnZ; ii) PGRP2+colistin: 6.7±0.5 for PAΔAG+pUCPAG and 6.9±2.05 for PAΔnZ+pUCPnZ. These results confirmed the causative relationship between each inactivation (*ampG* or *nagZ*) and the obtained phenotypes. In the case of complementation with pUCPAD in the triple amidase mutant, as can be observed in the Fig 3, the higher was the level of affectation of the double AmpD mutants, the less effective was the complementation. For instance, in the Fig 3A and 3B, the transformation with pUCPAD only restored the phenotype reaching the levels of double AmpD mutants, whereas in the Fig 3C and 3D, as the double amidase mutants were only slightly affected, the complementation was closer to the wild type levels.

Thus, our results show that, once the permeability barrier is overpassed, peptidoglycan recycling plays a major protective role against the PGRPs, particularly those classically considered bactericidal (PGRPs 1, 3, and 4), although the molecular basis for these observations still remains to be ellucidated. In this regard, some works have reported that PGRP1, 3 and 4 exert their bactericidal effect through the induction of a complex response based on the two-component system CpxA-CpxR, after the uniform binding of the PGRP to the outer gram-negative surface. It is not known whether after this binding, PGRP1, 3 and 4 also reach the peptidoglycan. [26,27,28,50,51]. These PGRPs are thought to cause a membrane depolarization and a subsequent stress in the periplasm sensed by the CpxA protein, and communicated to the cytoplasmic component CpxR, that would in turn up/downregulate the expression of several genes related with the envelope stress response. This would lead to a suicide through the inhibition of protein, RNA and DNA synthesis, as well as that of the cytosolic steps in the cell-wall synthesis [28,50],although the exact way how PGRP1, 3 and 4 activate this envelope stress response is unknown[52]. Thus, PGRP1, 3 and 4 seem to exert their bactericidal effects also affecting the peptidoglycan biology by dampening the initial steps of its anabolic pathways [50]. Then, in the absence of the first steps of cell-wall synthesis, the recycling of peptidoglycan would become essential to avoid the perturbation of the metabolism and of the cell-wall itself, and the derived activation of certain auto-lesive stress responses (likely mediated by the Cpx-like system [53]).

Some works have recently characterized the changes in peptidoglycan following the CpxA-CpxR response in *E*. *coli* [54,55], as the increase in the abundance of diaminopimelic acid (DAP)-DAP cross-links. Besides, it has been shown that during the Cpx activation, a certain level of protection is achieved against the exposure to β-lactams, probably through this kind of peptidoglycan modifications. However, it has been also found that Cpx over-activation levels lead to nocive consequences such as aberrant morphologies, increased susceptibility to β-lactams, and growth defects, all consistent with a loss of peptidoglycan homeostasis [55]. Considering these findings, it is tempting to especulate that PGRPs action could lead to an over-activation of Cpx response, causing loss of cell-wall homeostasis leading to bacterial death, wich would be consistent with our results. Thus, the novelty that can be deduced from our work is that when the permeability barrier is overpassed, the PGRP1, 3 and 4 drastically improve their bactericidal power likely thanks to a better access and binding to the peptidoglycan, and that once there, their antipseudomonal activity is greatly enhanced in a peptidoglycan recycling-defective background.

As commented above, the results for PGRP2 were slightly different, since its activity was higher in the triple amidase mutant than in the AmpG, and, especially, NagZ mutants, suggesting that peptidoglycan recycling is not the only player involved. The PGRPs were initially described as “peptidoglycan recognizing”, when first purified from the silkworm, based on their high affinity for peptidoglycan [56], but in humans only the PGRP2 is capable of hydrolyzing it [25,30,57,58]. Nonetheless, the PGRP2 has not been classically considered as a directly bactericidal protein [30] but some available data suggest that it may also behave as a cell-wall lytic and bactericidal protein in some models [48,50]. Either way, we have demonstrated for the first time that once overpassed the permeability barrier, PGRP2 shows high antipseudomonal activity, and that the mutants with impaired peptidoglycan recycling even show an enhanced susceptibility to it at different degrees. In this sense, the NagZ mutant is that with the lower degree of susceptibility increase to PGRP2, which is consistent with its less impaired peptidoglycan recycling: the pathways for stem peptide turnover would still be functional in this mutant, thanks to the entrance into the cytosol through the oligopeptide permease Opp [7]. Similarly to the case of lysozyme, these results suggest that the recycling of the stem-peptide, not affected in the NagZ mutant, may play a decisive protective role against the lytic aggresion driven by PGRP2. Nevertheless, PGRP2 may also contribute to activate the Cpx-like responses and derived effects, as the PGRP1, 3 and 4 likely do.

In summary, our data so far denote that for lysozyme, and to a lower extent for PGRP2, there are additional factors involved in the higher susceptibility of the triple amidase mutant, issue that will be addressed in next section.

### Impact of AmpC hyper-expression in the bactericidal activity of lysozyme and PGRPs

**Fig 4:**
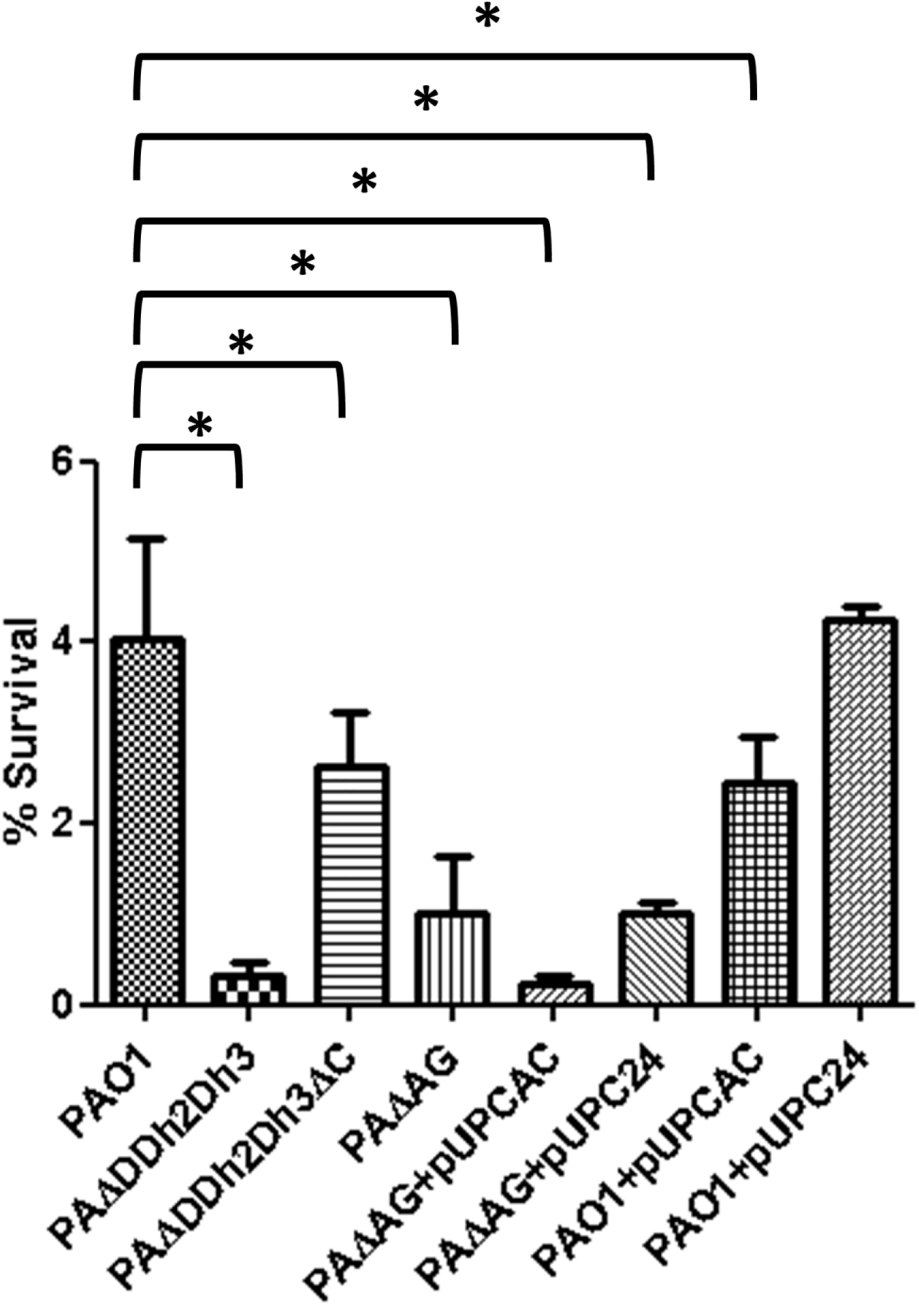
Bactericidal activity of lysozyme + colistin treatment in PAO1 and derived peptidoglycan recycling defective / AmpC hyper-expressing strains. The percentages of bacterial survival after treatments respect to the initial inoculum are shown; the bars represent the mean of 5 experiments and the error bars the SD. *Statistically significant, *P*<0.001 in the One-way ANOVA with post hoc Tukey’s multiple comparison test. PAΔDDh2Dh3: knockout mutant on *ampD*, *ampDh2* and *ampDh3* genes; PAΔDDh2Dh3ΔC: knockout mutant on *ampD*, *ampDh2*, *ampDh3* and *ampC* genes. PAΔAG: knockout mutant on *ampG*. pUCPAC: pUC18-based *Escherichia-Pseudomonas* shuttle vector containing PAO1 AmpC gene. pUCP24: empty pUC18-based *Escherichia-Pseudomonas* shuttle vector.

Given the results from our recent work [17] in which we demonstrated that the fitness and virulence impaired phenotype of the triple amidase mutant was specifically mediated by simultaneously blocking peptidoglycan recycling and overexpressing AmpC, we hypothesized that a similar principle could drive the susceptibility to lysozyme and PGRP2. Thus, a new set of mutants specifically designed to address this issue was tested, and the results for lysozyme plus colistin treatment are shown in Fig 4. Interestingly, knocking out AmpC in the triple amidase mutant greatly enhanced survival (PAΔDDh2Dh3ΔC was significantly more resistant than PAΔDDh2Dh3 to lysozyme+colistin treatment, approximately 10-fold, with *P*<0.001 in the One-way ANOVA with post hoc Tukey’s test). Meanwhile, overexpressing AmpC in the AmpG mutant significantly decreased its resistance [PAΔAG+pUCPAC showed a decreased survival (approximately 5-fold) in comparison with PAΔAG, with *P*<0.001]. Moreover, overexpressing AmpC in wild-type PAO1 (through the introduction of plasmid pUCPAC [17]) produced a very modest but significant reduction of survival compared to wild-type. Thus, as previously documented for fitness and virulence [17], the greatly increased susceptibility of the triple amidase mutant to lysozyme is mediated by simultaneously blocking peptidoglycan recycling and overexpressing AmpC.

**Fig 5:**
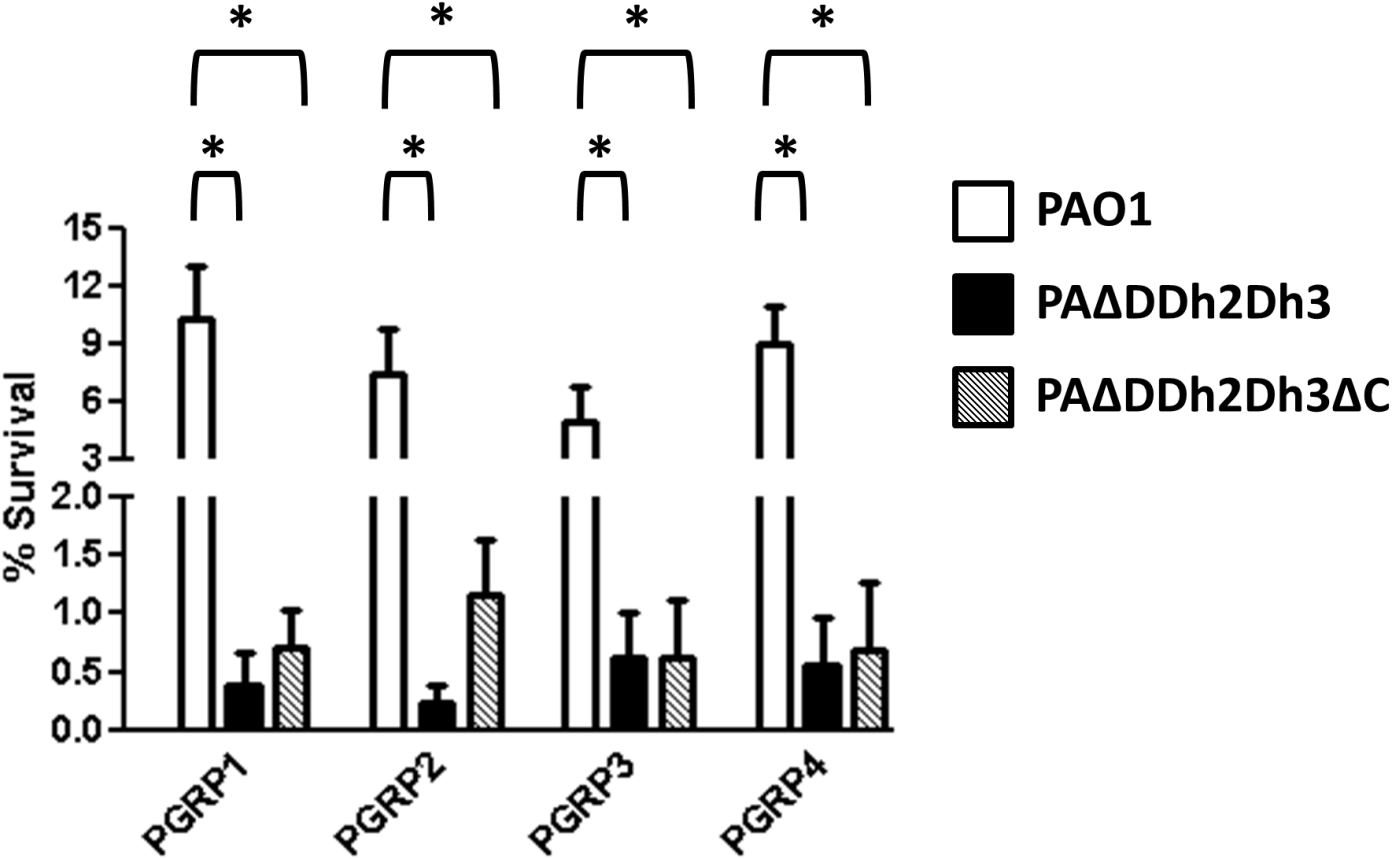
Bactericidal activity of PGRPs + colistin treatment in PAO1 and derived triple amidase mutants with / without AmpC deleted. The percentages of bacterial survival after treatments respect to the initial inoculum are shown. The bars represent the mean of 5 experiments and the error bars the SD; *Statistically significant, *P*< 0.001 in the One-way ANOVA with post hoc Tukey’s multiple comparison test. PAΔDDh2Dh3: knockout mutant on *ampD*, *ampDh2* and *ampDh3* genes; PAΔDDh2Dh3ΔC: knockout mutant on *ampD*, *ampDh2*, *ampDh3* and *ampC* genes.

To assess if the AmpC overexpression could have any impact as well on the susceptibility of *P*. *aeruginosa* to PGRPs, we analyzed if deleting the *ampC* gene in the triple amidase mutant increased survival. As shown in Fig 5, knocking out AmpC did not mitigate the increased susceptibility of the triple amidase mutant to PGRP1, 3 and 4, confirming that the phenotype is produced exclusively by the impairment of peptidoglycan recycling. On the other hand, survival after treatment with PGRP2 was notably increased (although without statistic significance and still far from wild-type levels) in the triple amidase-AmpC mutant. Thus, once more, a similar trend between lysozyme and PGRP2 was documented.

**Fig 6:**
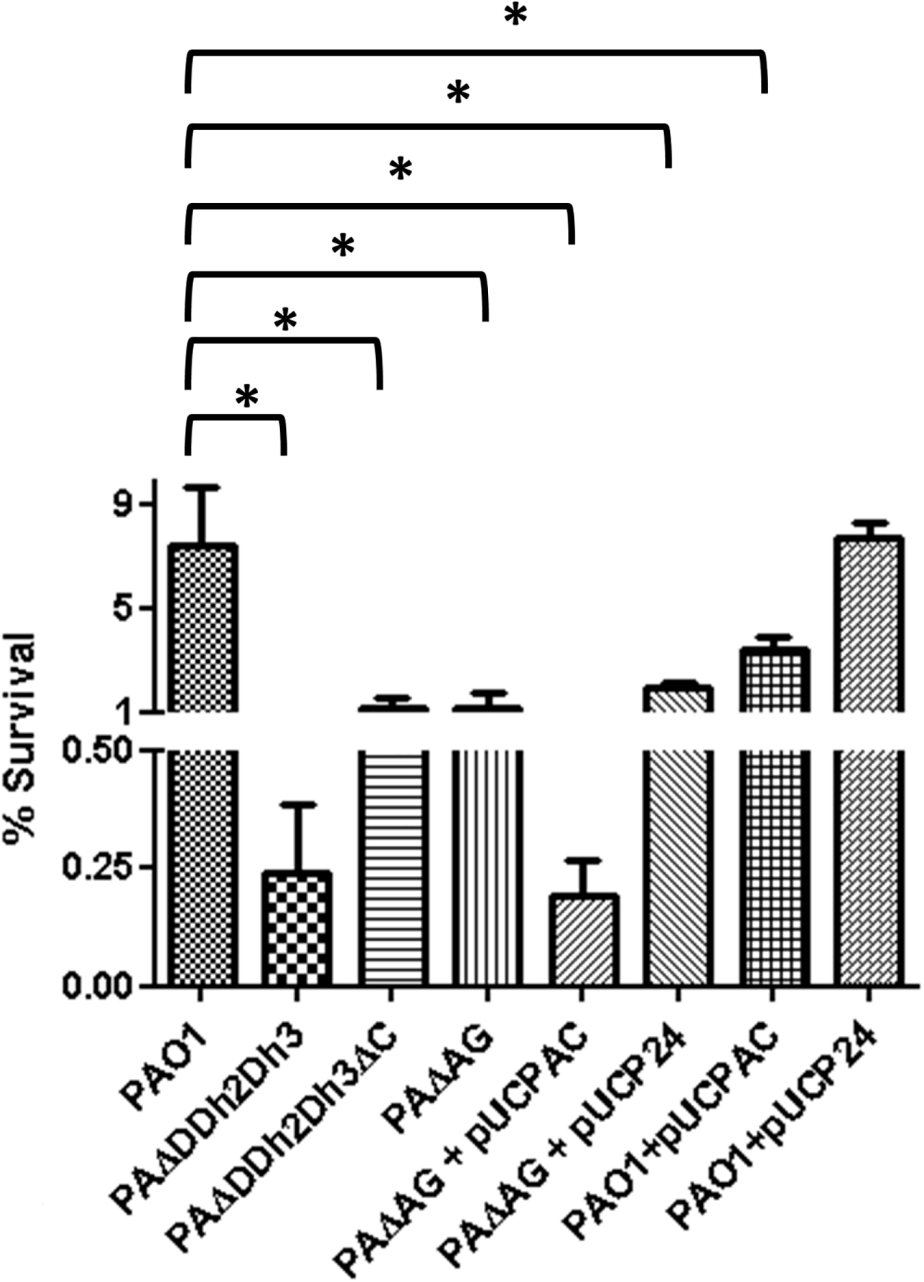
Bactericidal activity of PGRP2 + colistin treatment in PAO1 and derived peptidoglycan recycling defective / AmpC hyper-expressing strains. The percentages of bacterial survival after treatments respect to the initial inoculum are shown. The bars represent the mean of 5 experiments and the error bars the SD; *Statistically significant, *P*<0.001 in the One-way ANOVA with post hoc Tukey’s multiple comparison test. PAΔDDh2Dh3: knockout mutant on *ampD*, *ampDh2* and *ampDh3* genes; PAΔDDh2Dh3ΔC: knockout mutant on *ampD*, *ampDh2*, *ampDh3* and *ampC* genes. PAΔAG: knockout mutant on *ampG*. pUCPAC: pUC18-based *Escherichia-Pseudomonas* shuttle vector containing PAO1 AmpC gene. pUCP24: empty pUC18-based *Escherichia-Pseudomonas* shuttle vector.

To gain insights into the impact of AmpC hyperexpression on hypersusceptibility to PGRP2, we analyzed the profiles of resistance on additional strains, shown in Fig 6. As can be observed, the hyper-expression of AmpC *per se* produced a modest but significant effect, since the percentage of survival of PAO1 (7.5%) was decreased by approx. 2-fold in the PAO1 + pUCPAC derivative (3.4%). Moreover, the inactivation of AmpC in the triple amidase background increased survival rates from 0.25% to 1.2%, although wild-type PAO1 levels were not reached. Likewise, the overexpression of AmpC in the peptidoglycan recycling defective mutant (PAΔG + pUCPAC) decreased survival to PAΔDDh2Dh3 levels (circa 0.25%). Thus, the profiles of hypersusceptibility against lysozyme and PGRP2 were quite similar: the recycling blockade *per se* entailed a dramatic decrease in survival rates, but the simultaneous overexpression of AmpC further enhanced the antipseudomonal activity. The potential basis for these results will be discussed in the next section.

### Quantitative alterations in peptidoglycan composition associated with the hyper-susceptibility to lysozyme and PGRP2

We next examined whether the increased susceptibility of the peptidoglycan recycling deficient mutants to lysozyme and PGRPs, and the further contribution of AmpC overexpression in the case of lysozyme and PGRP2, could be correlated with quantitative and/or qualitave modifications of the cell-wall.

S2 Fig shows the chromatograms derived from the HPLC analysis of the muropeptides from our set of strains with defective peptidoglycan recycling and/or AmpC hyper-expression. As can be observed, the differences between all the chromatograms seem to be minimal, if any. Moreover, as shown in S1 Table, the absence of significant differences among the peptidoglycans of the studied strains was confirmed through the analysis of the typical muropeptide-derived parameters [43], namely: i) relative abundance of muropeptides monomers, dimers, trimers, muropeptides having DAP-DAP peptide bridges, muropeptides bound to Braun's lipoprotein, muropeptides having anhydro-1,6-anhydromuramic acid and muropeptides having a pentapeptide stem; ii) degree of cross-linking; and iii) average number of disaccharide units per glycan strand. Thus, the results from HPLC suggested that the basis for the enhanced susceptibility of the mentioned mutants to lysozyme / PGRPs was not apparently due to a qualitative modification of cell-wall structure.

Nevertheless, in order to rule out if some qualitative differences among the strains could have gone unnoticed in the HPLC analysis, we also studied the response elicited on commercial cell lines intended for the study of the activation of peptidoglycan receptors (HEK-Blue hNOD). It is known that specific peptidoglycan-derived fragments and their modifications depending on the species or even the strains can differentially stimulate our specialized receptors, namely the NOD-like receptors NOD1 and NOD2 [58,59,60]. Thus, we studied the levels of activation of each NOD receptor caused by the peptidoglycans purified from PAO1 or the triple amidase mutant (as a model of a peptidoglycan recycling blocked/AmpC hyperproducing strain) in the HEK-Blue hNOD cultures, and additionally, those caused by heat inactivated bacteria, viable bacteria and cell free bacterial cultures supernatants (to assess if the peptidoglycan fragments released to extracellular medium could also constitute a hint to infer biochemical particularities of each peptidoglycan). We also included the PA14 strain and its derived triple amidase mutant, to fully discard the differences in NOD stimulation when comparing wild-type and mutant strains. As can be observed in S3 and S4 Figs, no statistically significant differences [always comparing each wildtype strain with its derived mutant] in the activation of NOD1 or NOD2 receptors, could be detected when stimulating the HEK cells with the mentioned stimuli. It has been described that modifications in peptidoglycan composition trigger different levels of activation of NOD receptors, eliciting differential levels of inflammation [61]. Thus, our results suggest the absence of chemical modifications in the peptidoglycan composition of our mutants, responsible for the enhanced susceptibility to lysozyme or PGRPs treatments, and important enough to differentially stimulate the NODs receptors. These facts are in agreement with those of our recent work in which the peptidoglycans from PAO1 and derived amidase mutants did not show differential inflammatory capacities over A549 cell cultures, in terms of IL-8 release [17].

Thus, given the absence of biochemical qualitative differences in the peptidoglycans composition between wild type and mutants according to NOD receptors stimulation and HPLC parameters results, we decided to determine the total amount of peptidoglycan, as a possible additional explanation for the reduced resistance of the previously mentioned mutants against lysozyme / PGRPs. As can be observed in Table 2, the meso-diaminopimelic acid (mDAP) titration (an indirect method of peptidoglycan quantification [45]), revealed that only the strains PAΔDDh2Dh3 and PAΔAG + pUCPCAC showed a statistically significant reduction, of circa 30%, in the normalized quantity of peptidoglycan. The mDAP amount was restored to PAO1 levels by knocking out *ampC* in the triple *ampD* mutant, as well as through the transformation with pUCPAD (containing the wild-type *ampD*). Moreover, the double amidase mutants did not show any reduction in the mDAP titration, as well as the PAΔAG and PAΔnZ strains. Thus, the profile of reduced amount of peptidoglycan in PAΔDDh2Dh3 and PAΔAG + pUCPAC correlates with their increased susceptibility to lysozyme and PGRP2 treatments, and with the need of both events occurring simultaneously to provide the phenotype. How AmpC overexpression contributes to the phenotype remains to be explored, but the hypothesis to be addressed range from the simple energetic burden of producing large amounts of the enzyme to a direct effect of the β-lactamase on cell-wall physiology, perhaps due to a suggested residual DD-peptidase activity reminiscent of its potential PBP ascendance [17,62,63].

**Table 2:** Peptidoglycan (PGN) quantification through the titration of meso-diaminopimelic acid (mDAP).

Strain	mDAP amount^a^	Relative PGN amount with regards to PAO1
PAO1	215.6 ± 17.5	1
PAΔDDh2Dh3	149.1 ± 9.1*	0.69*
PAΔDDh2Dh3 + pUCPAD	227.6 ± 15.4	1.05
PAΔDDh2	208.9 ± 4.2	0.97
PAΔDDh3	196.5 ± 5.0	0.91
PAΔDh2Dh3	220.3 ± 10.1	1.02
PAΔDDh2Dh3ΔC	193.1 ± 13.9	0.89
PAΔAG	219.5 ± 16.1	1.02
PAΔAG + pUCPCAC	157.5 ± 22.1*	0.73*
PAΔAG + pUCPC24	191.3 ± 12.0	0.89
PAΔnZ	211.5 ± 14.9	0.98

^a^The peptidoglycan from selected strains was quantified by means of the titration of mDAP concentration, following the protocols described in Materials & Methods section. Bacterial cultures previously adjusted to ensure the same number of cells on each.The concentration of muropeptides was calculated using a mDAP standard curve, and the given value is the amount of mDAP (μg) purified from a 250 mL exponential culture adjusted to DO_600_ = 0.8. The results represent the mean ± SD from three different quantifications per strain.

*Statistically significant: *P*<0.001 in the One-way ANOVA with post hoc Tukey’s multiple comparison test (with regards to PAO1 strain). PAΔDDh2Dh3: knockout mutant on *ampD*, *ampDh2* and *ampDh3* genes; PAΔDDh2Dh3ΔC: knockout mutant on *ampD*, *ampDh2*, *ampDh3* and *ampC* genes PAΔnZ: knockout mutant on *nagZ*. PAΔAG: knockout mutant on *ampG*. pUCPAD: pUC18-based *Escherichia-Pseudomonas* shuttle vector containing PAO1 AmpD gene. pUCPAC: pUC18-based *Escherichia-Pseudomonas* shuttle vector containing PAO1 AmpC gene. pUCP24: empty pUC18-based *Escherichia-Pseudomonas* shuttle vector.

Although it has been recently proposed that the bactericidal power of lysozyme could be also independent of its lytic activity, and then related to membrane perturbation capacity [34,49], in the permeabilizing conditions of our experiments, it seems plausible that the prevailing mode of action of lysozyme was the direct lysis. Besides, although several contradictory models regarding the architecture of gram-negative cell-walls have been proposed, a planar model with specific regions of multilayered peptidoglycan is generally accepted, as is the idea of a relatively thinner *P*. *aerguginosa* peptidoglycan in comparison with other gram-negatives [64,65]. Thus a reduction of circa 30% on its amount might not be neglectable, mainly if affecting the cited multilayered peptidoglycan areas, likely key to maintain the capacity to counteract the osmotic pressure [64,65].

Meanwhile, although the seric PGRP2 was initially described as a scavenger protein intended to reduce the inflammatory capacity of peptidoglycan fragments by hydrolyzing them [29,30], several studies with different animal models have shown contradictory results regarding its pro- or anti-inflammatory effects and the consequences on the outcome of the host [33,51,58, 66].Thus, besides this controversy on PGRP2 inflammation-regulatory role, some recent works also bestowe it with bactericidal power [48], thanks to its hydrolytic activity of the the peptidoglycan, although it was not classically considered as bacteriolytic [30]. With our work we clearly show that PGRP2 has a high antipseudomonal power when combined with subinhibitory concentrations of colistin. We have shown that the recycling blockage, and regardless of AmpC de-repression, determines a dramatic increase of the anti-pseudomonal power of all four PGRPs, including PGRP2. These circumstances would suggest that PGRP2 triggers autolesive responses similar to those caused by PGRP1, 3 and 4, presumably through the direct binding to the cell-wall and the Cpx-like system. In addition, the reduction of the total peptidoglycan amount caused by the simultaneous blockage of peptidoglycan recycling and the AmpC hyper-expression appears to cause a further increased PGRP2 susceptibility, perhaps denoting a lytic activity similar to that of lysozyme.

## Conclusions

Throughout this study we show that *P*. *aeruginosa* is highly resistant to lysozyme and PGRPs mainly thanks to its outer membrane permeability barrier, and that the lysozyme inhibitor proteins MliC, IvyP1 and IvyP2, do not seem to play a major protective role even in permeabilizing conditions. Moreover, we show that once the permeability barrier is overcome, the bactericidal power of lysozyme and PGRPs is dramatically increased in *P*. *aeruginosa* suggesting that direct peptidoglycan binding may play a major role in their activities. This is the first report demonstrating synergy between colistin and peptidoglycan-targeting immune proteins delineating new strategies for anti-pseudomonal therapies. Thus, our results align with recent works starting to propose engineered lysozyme and synergies with this innate immune protein as potential anti-pseudomonal treatments for the future [67,68].

We additionally show that peptidoglycan recycling seems to play a key protective role against lysozyme and all four PGRPs activity in *P*. *aeruginosa* once the permeability barrier is broken. Furthermore, we show that in the case of lysozyme and PGRP2, highest bactericidal activity is achieved by simultaneously blocking peptidoglycan recycling and overexpressing AmpC. Indeed, this phenotype was found to be associated with a significant (circa 30%) reduction of total amount of peptidoglycan per cell, which may result in an increased susceptibility to the lytic activity of lysozyme and PGRP2.

Taken together our results determine a major step forward for understanding the biology of *P*. *aeruginosa* to resist the action of immune proteins targeting the peptidoglycan, revealing weak points potentially exploitable as targets that should be helpful for guiding the development of future strategies to fight infections, simultaneously targeting β–lactam resistance, cell-wall metabolism and virulence. Thus, the recently described NagZ inhibitors [9,10,69], together with those being searched for against AmpG [70], when used together with permeabilizing agents such as colistin, could be useful weapons not only to revert the AmpC-driven β-lactam resistance [9,10], but also to make *P*. *aeruginosa* more susceptible to innate immune weapons such as lysozyme or PGRPs.

## Supporting information

S1 Fig Bactericidal activity of lysozyme (25 μg/mL) or colistin (0.1 μg/mL) treatments in PAO1 and derived knockout mutants, in the lysozyme assay buffer. The percentages of bacterial survival after treatments respect to the initial inoculum are shown; The bars represent the mean of 5 experiments and the error bars the SD. *Statistically significant, *P*<0.05 in the One-way ANOVA test. PAΔDDh2Dh3: knockout mutant on *ampD*, *ampDh2* and *ampDh3* genes; PAΔnZ: knockout mutant on *nagZ*. PAΔAG: knockout mutant on *ampG*. pUCPAD: pUC18-based *Escherichia-Pseudomonas* shuttle vector containing PAO1 AmpD gene.
(TIF)

S2 Fig High-performance liquid chromatograms of peptidoglycan muropeptides of the indicated strains. Each series displays peaks corresponding to the common muropeptides in peptidoglycan of each strain. Each peak corresponds to a typical muropeptide whose name is indicated at the top. M3, disaccharide tripeptide; M4, disaccharide tetrapeptide; D43, cross-linked dimer of disaccharide tetrapeptide-disaccharide tripeptide; D44, cross-linked dimer of disaccharide tetrapeptide-disaccharide tetrapeptide; D43L, cross-linked dimer of disaccharide tetrapeptide-disaccharide tripeptide bound to lipoprotein; T444, cross-linked trimer of disaccharide tetrapeptide-disaccharide tetrapeptide-disaccharide tetrapeptide; D44N have the same structures as muropeptides D44, but with anhydro-N-acetylmuramic acid instead of N-acetylmuramic acid. Each disaccharide is composed of N-acetylglucosamine and N-acetylmuramic acid. PAΔDDh2Dh3: knockout mutant on *ampD*, *ampDh2* and *ampDh3* genes; PAΔDDh2Dh3ΔC: knockout mutant on *ampD*, *ampDh2*, *ampDh3* and *ampC* genes PAΔnZ: knockout mutant on *nagZ*. PAΔAG: knockout mutant on *ampG*. pUCPAC: pUC18-based *Escherichia-Pseudomonas* shuttle vector containing PAO1 AmpC gene.
(TIF)

S3 Fig Activation of HEK-Blue hNOD1 cells with PAO1 and PA14-derived strains. 620 nm absorbance (proportional to NOD1 activation) after 20 h of stimulation with: A) heat-inactivated bacteria, MOI 1000; B) viable bacteria, MOI 250; C) cell-free supernatants (10% in detection medium) and D) purified peptidoglycans (PGN), 1 μg/well. 0.2 μg of C12-iE-DAP per well were used as positive control, whereas PBS was used as negative control. The results represent the mean ± SD from seven wells of HEK-Blue cells proceeding from three independent plates. *Statistically significant, *P*< 0.05 in the Student’s t-test. PAΔDDh2Dh3: PAO1 knockout mutant on *ampD*, *ampDh2* and *ampDh3* genes. PA14ΔDDh2Dh3: PA14 knockout mutant on *ampD*, *ampDh2* and *ampDh3* genes.
(TIF)

S4 Fig Activation of HEK-Blue hNOD2 cells with PAO1 and PA14-derived strains. 620 nm absorbance (proportional to NOD2 activation) after 20h of stimulation with: A) heat-inactivated bacteria MOI 500, B) viable bacteria, MOI 250; C) cell-free supernatants (10% in detection medium) and D) purified PGNs, 0.25 μg/well. 2 μg/mL of MDP were used as positive control, whereas PBS was used as negative control. The results represent the mean ± SD from seven wells of HEK-Blue cells proceeding from three independent plates. *Statistically significant, *P* < 0.05 in the Student’s t-test. PAΔDDh2Dh3: knockout mutant on *ampD*, *ampDh2* and *ampDh3* genes. PA14ΔDDh2Dh3: PA14 knockout mutant on *ampD*, *ampDh2* and *ampDh3* genes.
(TIF)

S1 Table HPLC analysis of muropeptides prepared from the peptidoglycan of the PAO1 and derived knockout mutants. (DOCX)

S2 Table Strains and plasmids used in this work. (DOCX)
