# Supplementary figures and images for: Targeting the permeability barrier and peptidoglycan recycling pathways to disarm Pseudomonas aeruginosa against the innate immune system
Source: PLoS One. 2017 Jul 25;12(7):e0181932. doi: 10.1371/journal.pone.0181932 (PMC5526577; doi:10.1371/journal.pone.0181932)

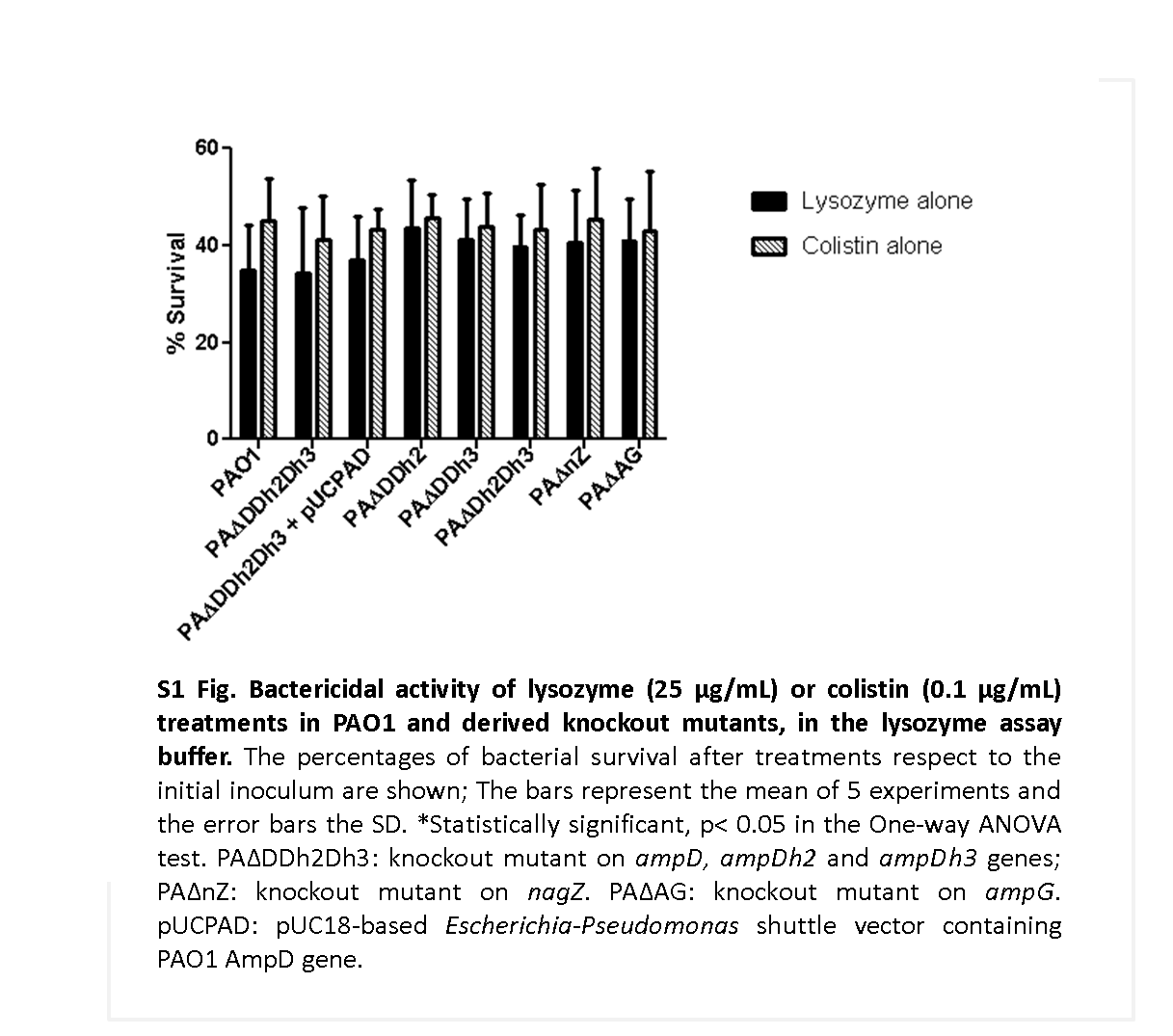

Supplement: S1 Fig — The percentages of bacterial survival after treatments respect to the initial inoculum are shown; The bars represent the mean of 5 experiments and the error bars the SD. *Statistically significant, P<0.05 in the One-way ANOVA test. PAΔDDh2Dh3: knockout mutant on ampD, ampDh2 and ampDh3 genes; PAΔnZ: knockout mutant on nagZ. PAΔAG: knockout mutant on ampG. pUCPAD: pUC18-based Escherichia-Pseudomonas shuttle vector containing PAO1 AmpD gene. (TIF) [file pone.0181932.s001.tif]

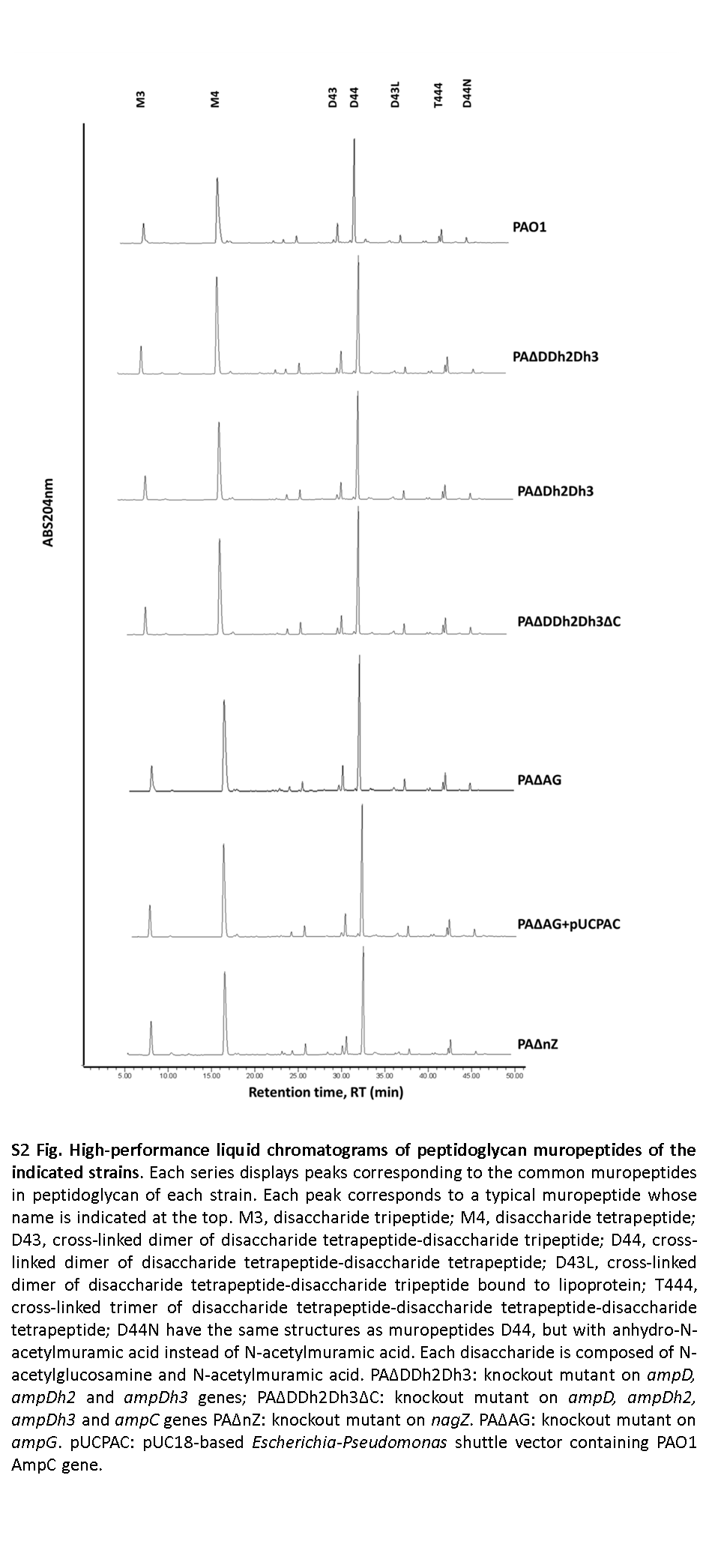

Supplement: S2 Fig — Each series displays peaks corresponding to the common muropeptides in peptidoglycan of each strain. Each peak corresponds to a typical muropeptide whose name is indicated at the top. M3, disaccharide tripeptide; M4, disaccharide tetrapeptide; D43, cross-linked dimer of disaccharide tetrapeptide-disaccharide tripeptide; D44, cross-linked dimer of disaccharide tetrapeptide-disaccharide tetrapeptide; D43L, cross-linked dimer of disaccharide tetrapeptide-disaccharide tripeptide bound to lipoprotein; T444, cross-linked trimer of disaccharide tetrapeptide-disaccharide tetrapeptide-disaccharide tetrapeptide; D44N have the same structures as muropeptides D44, but with anhydro-N-acetylmuramic acid instead of N-acetylmuramic acid. Each disaccharide is composed of N-acetylglucosamine and N-acetylmuramic acid. PAΔDDh2Dh3: knockout mutant on ampD, ampDh2 and ampDh3 genes; PAΔDDh2Dh3ΔC: knockout mutant on ampD, ampDh2, ampDh3 and ampC genes PAΔnZ: knockout mutant on nagZ. PAΔAG: knockout mutant on ampG. pUCPAC: pUC18-based Escherichia-Pseudomonas shuttle vector containing PAO1 AmpC gene. (TIF) [file pone.0181932.s002.tif]

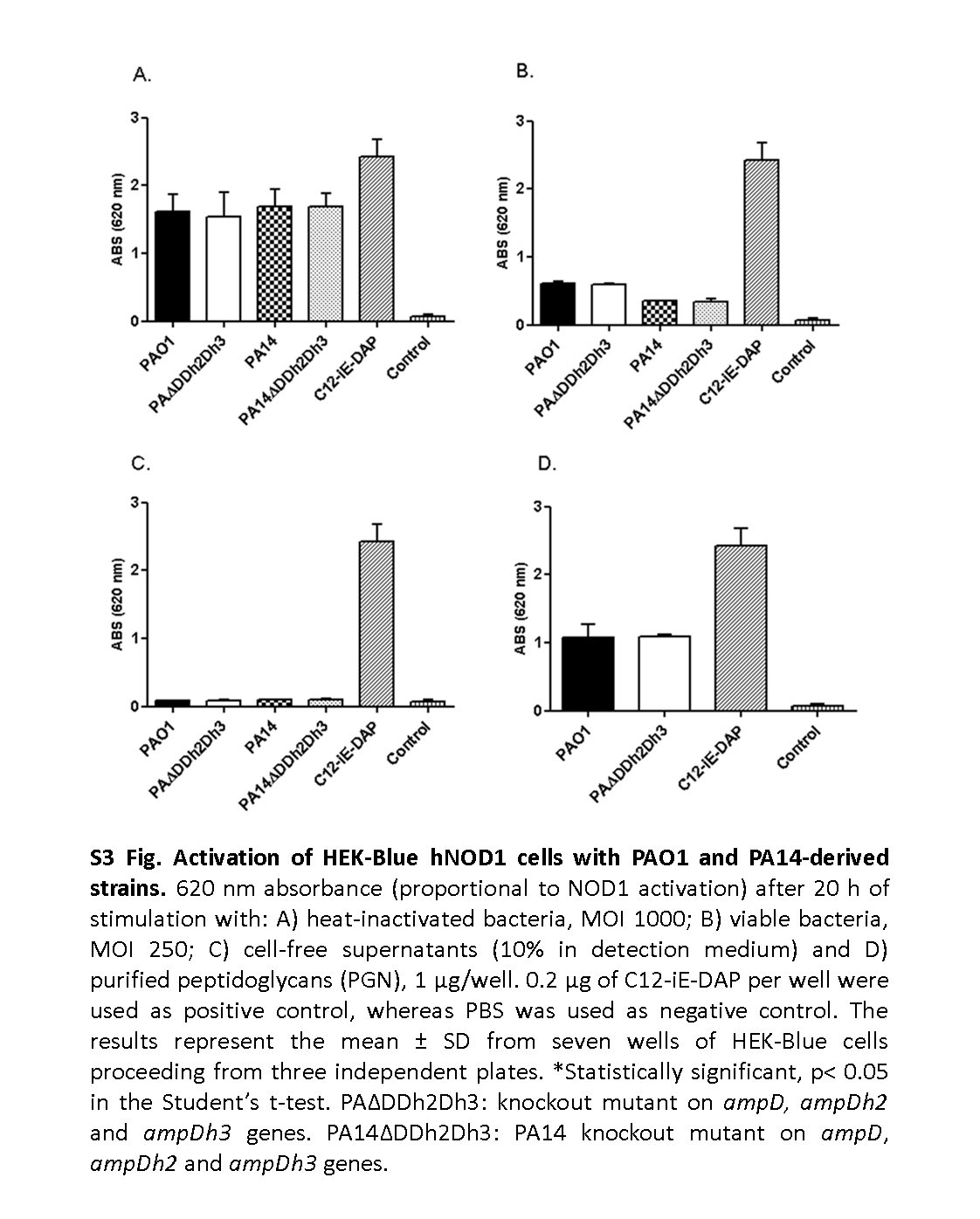

Supplement: S3 Fig — 620 nm absorbance (proportional to NOD1 activation) after 20 h of stimulation with: A) heat-inactivated bacteria, MOI 1000; B) viable bacteria, MOI 250; C) cell-free supernatants (10% in detection medium) and D) purified peptidoglycans (PGN), 1 μg/well. 0.2 μg of C12-iE-DAP per well were used as positive control, whereas PBS was used as negative control. The results represent the mean ± SD from seven wells of HEK-Blue cells proceeding from three independent plates. *Statistically significant, P< 0.05 in the Student’s t-test. PAΔDDh2Dh3: PAO1 knockout mutant on ampD, ampDh2 and ampDh3 genes. PA14ΔDDh2Dh3: PA14 knockout mutant on ampD, ampDh2 and ampDh3 genes. (TIF) [file pone.0181932.s003.tif]

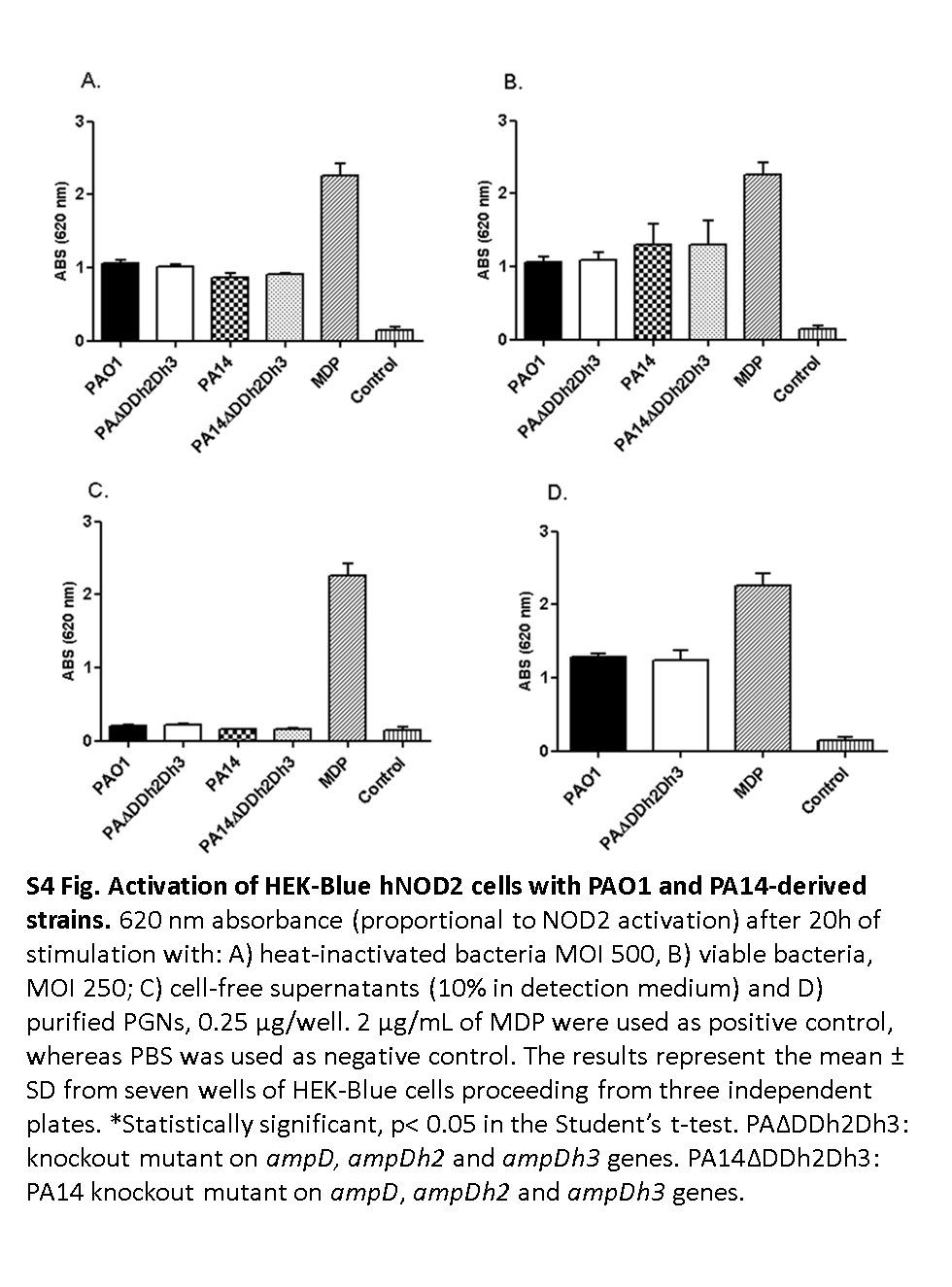

Supplement: S4 Fig — 620 nm absorbance (proportional to NOD2 activation) after 20h of stimulation with: A) heat-inactivated bacteria MOI 500, B) viable bacteria, MOI 250; C) cell-free supernatants (10% in detection medium) and D) purified PGNs, 0.25 μg/well. 2 μg/mL of MDP were used as positive control, whereas PBS was used as negative control. The results represent the mean ± SD from seven wells of HEK-Blue cells proceeding from three independent plates. *Statistically significant, P < 0.05 in the Student’s t-test. PAΔDDh2Dh3: knockout mutant on ampD, ampDh2 and ampDh3 genes. PA14ΔDDh2Dh3: PA14 knockout mutant on ampD, ampDh2 and ampDh3 genes. (TIF) [file pone.0181932.s004.tif]
