# Supplementary material for: Targeting the permeability barrier and peptidoglycan recycling pathways to disarm Pseudomonas aeruginosa against the innate immune system
Source: PLoS One. 2017 Jul 25;12(7):e0181932. doi: 10.1371/journal.pone.0181932 (PMC5526577; doi:10.1371/journal.pone.0181932)
Supplement: S1 Table — (DOCX) [file pone.0181932.s005.docx]

**Table S1.** HPLC analysis of muropeptides prepared from the peptidoglycan of the PAO1 and derived knockout mutants.

| Strain | Relative abundance (mol%) of muropeptide^a^ | | | | | | | Cross-link^b^ | Average glycan chain length^c^ |
| --- | --- | --- | --- | --- | --- | --- | --- | --- | --- |
|  | Mono | Di | Tri | DD | Lpp | Anh | Pent |  |  |
| PAO1 | 61.8 ± 3.1 | 34.4 ± 2.7 | 2.9 ± 0.5 | 0 | 6.2 ± 1.8 | 1.6 ± 0.4 | 0 | 42.5 ± 2.6 | 58.5 ± 10.1 |
| PA∆DDh2Dh3 | 63.3 ± 2.1 | 33.2 ± 2.2 | 2.8 ± 0.7 | 0 | 6.2 ± 1.4 | 1.3 ± 0.47 | 0 | 39.2 ± 2.9 | 65.6 ± 12.1 |
| PA∆Dh2Dh3 | 62.0 ± 5.7 | 33.5 ± 4.9 | 3.2 ± 0.8 | 0 | 6.0 ± 1.8 | 1.3 ± 0.7 | 0 | 41.0 ± 5.6 | 67.3 ± 11.1 |
| PA∆DDh2Dh3∆C | 62.1 ± 2.1 | 34.2 ± 1.9 | 2.8 ± 0.5 | 0 | 5.1 ± 1.5 | 1.4 ± 0.4 | 0 | 39.25 ± 2 | 63.7 ± 15.3 |
| PA∆AG | 64.3 ± 0.5 | 32.5 ± 0.7 | 2.5 ± 0.1 | 0 | 4.4 ±0.0 | 1.1 ± 0.1 | 0 | 41.6 ± 8.3 | 68.3 ± 9.2 |
| PA∆AG + pUCPAC | 63.9 ± 3.3 | 33.9 ± 5.2 | 2.8 ± 0.7 | 0 | 5.3 ± 1.1 | 1.3 ± 0.3 | 0 | 39.4 ± 5.7 | 66.1 ± 7.7 |
| PA∆nZ | 61.1 ± 4.1 | 34.5 ± 3.1 | 3.0 ± 0.5 | 0 | 5.1 ± 1.1 | 1.45 ± 0.1 | 0 | 41.5 ± 3.9 | 61.3 ± 8.9 |

^a^Molar fraction (mol%) of each muropeptide relative to the total content. Mono, monomers; Di, dimers; Tri, trimers; DD, muropeptides having Dap-Dap peptide bridges; Lpp, muropeptides bound to C-terminal Arg-Lys dipeptide of Braun's lipoprotein; Anh, muropeptides having anhydro-1,6-anhydromuramic acid; Pent, muropeptides having a pentapeptide stem. The values represent the mean of data from three independent HPLCs analysis per strain ± SD. ^b^Cross-link, degree of peptidoglycan cross-linking (percentage). ^c^The average number of disaccharide units is shown. *Statistically significant, p< 0.05 in the One-way ANOVA test. PAΔDDh2Dh3: knockout mutant on *ampD*, *ampDh2* and *ampDh3* genes; PAΔDDh2Dh3∆C: knockout mutant on *ampD*, *ampDh2*, *ampDh3* and *ampC* genes. PAΔnZ: knockout mutant on *nagZ*. PAΔAG: knockout mutant on *ampG*. pUCPAC: pUC18-based *Escherichia-Pseudomonas* shuttle vector containing PAO1 AmpC gene.
