# Supplementary material for: Targeting the permeability barrier and peptidoglycan recycling pathways to disarm Pseudomonas aeruginosa against the innate immune system
Source: PLoS One. 2017 Jul 25;12(7):e0181932. doi: 10.1371/journal.pone.0181932 (PMC5526577; doi:10.1371/journal.pone.0181932)
Supplement: S2 Table — (DOCX) [file pone.0181932.s006.docx]

**Table S2**. Strains and plasmids used in this work

| **Strain or**  **Plasmid** | **Genotype/relevant characteristic(s)** | **Reference or source** |
| --- | --- | --- |
| *P. aeruginosa* |  |  |
| PAO1 | Completely sequenced reference strain | Stover et al, 2000 |
| PAΔD | PAO1 ΔampD::*lox*; AmpD is an N-acetyl-anhydromuramyl–L-alanine amidase involved in peptidoglycan recycling; negative regulator of AmpC expression | Juan et al, 2006 |
| PAΔDh2 | PAO1 ΔampDh2::*lox*; AmpDh2 is an additional AmpD homologue of *P. aeruginosa* | Juan et al, 2006 |
| PAΔDh3 | PAO1 ΔampDh3::*lox*; AmpDh3 is an additional AmpD homologue of *P. aeruginosa* | Juan et al, 2006 |
| PAΔDh2Dh3 | PAO1 ΔampDh2::*lox* ΔampDh3::lox | Juan et al, 2006 |
| PAΔDDh2 | PAO1 ΔampD::*lox* ΔampDh2::*lox* | Juan et al, 2006 |
| PAΔDDh3 | PAO1 ΔampD::*lox* ΔampDh3::*lox* | Juan et al, 2006 |
| PAΔDDh2Dh3 | PAO1 ΔampD::*lox* ΔampDh2::*lox* ΔampDh3::*lox*  Mutant derepressed for AmpC production | Juan et al, 2006 |
| PAΔDDh2Dh3∆AC | PAO1 ΔampD::*lox* ΔampDh2::*lox* ΔampDh3::lox ΔampC::*lox*; *ampC* encodes the chromosomal cephalosporinase of *P. aeruginosa*. | Moya et al, 2008 |
| PAΔAG | PAO1 ΔampG::*lox; ampG* encodes the specific permease allowing the entry of peptidoglycan (PGN) derivatives into the cytosol of *P. aeruginosa*. | Zamorano et al, 2011 |
| PAΔnZ | PAO1 Δ*nagZ::lox*; *nagZ* encodes the β-N-acetylglucosaminidase essential to generate the 1,6-anhydromuropeptides which are thought to induce AmpC production. | Zamorano et al, 2010 |
| PAΔdacBΔD | PAO1 ΔdacB::*lox* ΔampD::*lox;*  Mutant derepressed for AmpC production | Moya et al, 2009 |
| PA14 | Completely sequenced reference strain | Lee et al, 2006 |
| PA14ΔDDh2Dh3 | PA14 ΔampD::lox ΔampDh2::lox ΔampDh3::lox; mutant derepressed for AmpC production | Pérez-Gallego et al, 2016 |
| PA14∆MliC | MAR2xT7 transposon insertion mutant in PA14_53040 gene (*mliC*); Gm^r^ | Liberati et al, 2006 |
| PA14∆IvyP1 | MAR2xT7 transposon insertion mutant in PA14_13420 gene (*IvyP1*); Gm^r^ | Liberati et al, 2006 |
| PA14∆IvyP2 | MAR2xT7 transposon insertion mutant in PA14_72360 gene (*IvyP2*); Gm^r^ | Liberati et al, 2006 |
| **Plasmids** |  |  |
| pUCPAD | Gm^r^; pUC18-based *Escherichia-Pseudomonas* shuttle vector containing PAO1 AmpD gene | Juan et al, 2005 |
| pUCPAC | Gm^r^; pUC18-based *Escherichia-Pseudomonas* shuttle vector containing PAO1 AmpC gene | Cabot et al, 2014 |
| pUCPAG | Gm^r^; pUC18-based *Escherichia-Pseudomonas* shuttle vector containing PAO1 AmpG gene | Zamorano et al, 2011 |
| pUCPnZ | Gm^r^; pUC18-based *Escherichia-Pseudomonas* shuttle vector containing PAO1 NagZ gene | Asgarali et al, 2009 |
|  |  |  |

**REFERENCES FOR S2 Table:**

Stover CK, Pham XQ, Erwin AL, Mizoguchi SD, Warrener P, Hickey MJ, Brinkman FS, Hufnagle WO, Kowalik DJ, Lagrou M, Garber RL, Goltry L, Tolentino E, Westbrock-Wadman S, Yuan Y, Brody LL, Coulter SN, Folger KR, Kas A, Larbig K, Lim R, Smith K, Spencer D, Wong GK, Wu Z, Paulsen IT, Reizer J, Saier MH, Hancock RE, Lory S, Olson MV. 2000. Complete genome sequence of *Pseudomonas aeruginosa* PAO1, an opportunistic pathogen. Nature 406:959–964. PMID: 10984043. 20.

Juan C, Moyá B, Pérez JL, Oliver A. 2006. Stepwise upregulation of the Pseudomonas aeruginosa chromosomal cephalosporinase conferring high-level beta-lactam resistance involves three AmpD homologues. Antimicrob Agents Chemother 50:1780-1787. PMID: 16641450.

Moya B, Juan C, Albertí S, Pérez JL, Oliver A. 2008. Benefit of having multiple ampD genes for acquiring beta-lactam resistance without losing fitness and virulence in Pseudomonas aeruginosa. Antimicrob Agents Chemother 52: 3694-3700. PMID: 18644952

Zamorano L, Reeve TM, Juan C, Moyá B, Cabot G, Vocadlo DJ, Mark BL, Oliver A .2011. AmpG inactivation restores susceptibility of pan-beta-lactam-resistant Pseudomonas aeruginosa clinical strains. Antimicrob Agents Chemother 55:1990-1996. PMID: 21357303.

Zamorano L, Reeve TM, Deng L, Juan C, Moyá B, Cabot G, Vocadlo DJ, Mark BL, Oliver A. 2010 NagZ inactivation prevents and reverts beta-lactam resistance, driven by AmpD and PBP 4 mutations, in Pseudomonas aeruginosa. Antimicrob Agents Chemother. 54:3557-63. PMID: 20566764

Moya B, Dötsch A, Juan C, Blázquez J, Zamorano L, Haussler S, Oliver A. 2009. Beta-lactam resistance response triggered by inactivation of a nonessential penicillin-binding protein. PLoS Pathog 5: e1000353. PMID: 19325877.

Lee DG, Urbach JM, Wu G, Liberati NT, Feinbaum RL, Miyata S, Diggins LT, He J, Saucier M, Déziel E, Friedman L, Li L, Grills G, Montgomery K, Kucherlapati R, Rahme LG, Ausubel FM. 2006. Genomic analysis reveals that Pseudomonas aeruginosa virulence is combinatorial. Genome Biol 7: R90. PMID: 17038190.

Pérez-Gallego M, Torrens G, Castillo-Vera J, Moya B, Zamorano L, Cabot G, Hultenby K, Albertí S, Mellroth P, Henriques-Normark B, Normark S, Oliver A, Juan C. Impact of AmpC Derepression on Fitness and Virulence: the Mechanism or the Pathway? MBio. 2016 Oct 25;7(5). pii: e01783-16. doi: 10.1128/mBio.01783-16. PMID: 27795406.

Cabot G, Bruchmann S, Mulet X, Zamorano L, Moyà B, Juan C, Haussler S, Oliver A. 2014. Pseudomonas aeruginosa ceftolozane-tazobactam resistance development requires multiple mutations leading to overexpression and structural modification of AmpC. Antimicrob Agents Chemother 58: 3091-3099. PMID: 24637685.

Juan C, Maciá MD, Gutiérrez O, Vidal C, Pérez JL, Oliver A. 2005. Molecular mechanisms of beta-lactam resistance mediated by AmpC hyperproduction in Pseudomonas aeruginosa clinical strains. Antimicrob Agents Chemother 49:4733-4738. PMID: 16251318.

Liberati NT, Urbach JM, Miyata S, Lee DG, Drenkard E, Wu G, Villanueva J, Wei T, Ausubel FM. 2006. An ordered, nonredundant library of Pseudomonas aeruginosa strain PA14 transposon insertion mutants. Proc Natl Acad Sci U S A. 21;103:2833-8. PMID: 16477005

Asgarali A, Stubbs K, Oliver A, Vocadlo D, Mark B. 2009. Inactivation of the Glycoside Hydrolase NagZ Attenuates Antipseudomonal β-Lactam Resistance in Pseudomonas aeruginosa. Antimicrob. Agents Chemother. 53: 2274-2282
